# The Gene Expression Landscape of Disease Genes

**DOI:** 10.1101/2024.06.20.24309121

**Published:** 2024-06-21

**Authors:** Judit García-González, Saul Garcia-Gonzalez, Lathan Liou, Paul F. O’Reilly

**Affiliations:** 1 Department of Genetics and Genomic Sciences, Icahn School of Medicine, Mount Sinai, New York City, NY 10029, USA; 2 Center for Excellence in Youth Education, Icahn School of Medicine, Mount Sinai, New York City, NY 10029, USA

## Abstract

Fine-mapping and gene-prioritisation techniques applied to the latest Genome-Wide Association Study (GWAS) results have prioritised hundreds of genes as causally associated with disease. Here we leverage these recently compiled lists of high-confidence causal genes to interrogate where in the body disease genes operate. Specifically, we combine GWAS summary statistics, gene prioritisation results and gene expression RNA-seq data from 46 tissues and 204 cell types in relation to 16 major diseases (including 8 cancers). In tissues and cell types with well-established relevance to the disease, the prioritised genes typically have higher absolute and relative (i.e. tissue/cell specific) expression compared to non-prioritised ‘control’ genes. Examples include brain tissues in psychiatric disorders (*P*-value < 1×10^−7^), microglia cells in Alzheimer’s Disease (*P*-value = 9.8×10^−3^) and colon mucosa in colorectal cancer (*P*-value < 1×10^−3^). We also observe significantly higher expression for disease genes in multiple tissues and cell types with no established links to the corresponding disease. While some of these results may be explained by cell types that span multiple tissues, such as macrophages in brain, blood, lung and spleen in relation to Alzheimer’s disease (*P*-values < 1×10^−3^), the cause for others is unclear and motivates further investigation that may provide novel insights into disease etiology. For example, mammary tissue in Type 2 Diabetes (*P*-value < 1×10^−7^); reproductive tissues such as breast, uterus, vagina, and prostate in Coronary Artery Disease (*P*-value < 1×10^−4^); and motor neurons in psychiatric disorders (*P*-value < 3×10^−4^). In the GTEx dataset, tissue type is the major predictor of gene expression but the contribution of each predictor (tissue, sample, subject, batch) varies widely among disease-associated genes. Finally, we highlight genes with the highest levels of gene expression in relevant tissues to guide functional follow-up studies. Our results could offer novel insights into the tissues and cells involved in disease initiation, inform drug target and delivery strategies, highlighting potential off-target effects, and exemplify the relative performance of different statistical tests for linking disease genes with tissue and cell type gene expression.

## Introduction

Genome-wide association studies (GWAS) for complex diseases have identified thousands of risk loci in the last two decades^1^. An important first step in translating GWAS findings into biological and clinical insights is to take broadly identified risk loci, incorporating associations across usually many genes due to linkage disequilibrium (LD), and interrogate them to pin-point the causal variants and genes. To identify causal disease genes, fine-mapping and gene prioritisation strategies have been developed^2^ and – only in recent years – lists of high-confidence causal genes for multiple diseases have been compiled^3–11^. Since (i) the probability of success in drug development increases with support for the relevant gene in GWAS^12^, and (ii) tissue-specific genes are more likely to become drug targets than broadly expressed genes^13–15^, profiling the gene expression of these disease-associated genes across multiple tissues and cell types could aid the development of new drugs^16,17^ and limit off-target effects^18,19^. However, a systematic characterisation of gene expression for GWAS prioritised genes has yet to be performed.

While understanding the specific cell types involved in disease and their spatial distribution has been of intense interest in recent years^20–25^, owing to the technological and computational advances of single-cell genomics (reviewed here^26^), we first focus here on identifying the relevant tissues in which disease-associated genes are expressed. Tissues known to be implicated in diseases serve as positive controls, essential to benchmark and optimise approaches that assess the relevance of cell and tissue types. Such positive controls are scarce for cell types^27,28^. Moreover, growing evidence highlights the intricate interconnections among the body’s various systems (nervous, immune, metabolic, hematopoietic, endocrine), suggesting that multiple tissues could typically be involved in disease^29–31^. After investigating tissues, we apply the same systematic approach at the higher resolution of the cell type.

**Figure 1.**
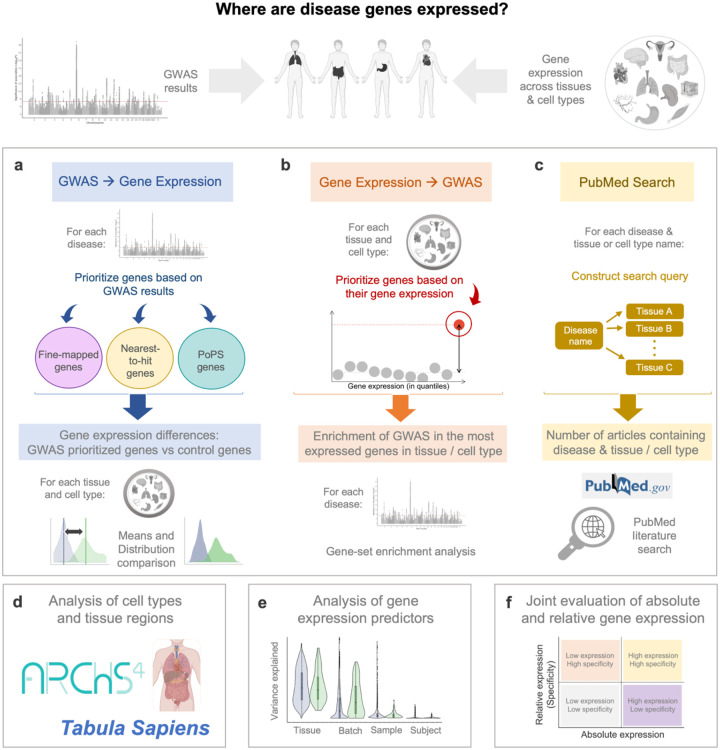
Overview of the study to characterise the gene expression features of genes associated to diseases. **a**, Approach using lists putatively causal genes, prioritized based on GWAS results. **b**, Approach using genes with the highest absolute expression or highest relative expression in each tissue and cell type. **c**, PubMed-based literature search to assess the tissues that are most often cited for each disease. **d,** The approaches in a-c were repeated for the cell types and tissue regions available in the ARCHS4 and Tabula Sapiens resources. **e,** The predictors of gene expression were analysed for each gene using the variance partition R package. **f**, For each gene, absolute and relative gene expression was evaluated. Figure partially created with BioRender.com.

In this study, we characterise the tissues and cell types in which disease-associated genes are expressed. Our primary analysis uses three alternative approaches that leverage RNA-seq and GWAS data: the first – that we call “*from GWAS to Gene Expression”* – interrogates whether genes prioritised as causal in the latest landmark GWAS of major diseases have distinct gene expression features from those of other protein-coding genes (Fig 1a). The second approach – that we call “*from Gene Expression to GWAS”* - examines whether high-expression genes are enriched for GWAS signal, as calculated by MAGMA^32^ (Fig 1b). In the third approach, we perform a systematic PubMed scan to assess the evidence for tissue-disease associations reported in the literature (Fig 1c). Contrasting results systematically across the three approaches allows for triangulation of results. Furthermore, we ensure the robustness of our study by employing three distinct definitions for disease-associated genes and utilising three different sets of control genes. We apply our testing framework to more than 200 cell types and tissue regions obtained from the ARCHS4^33^ and Tabula Sapiens^34^ resources (Fig 1d). We also characterise, for each individual gene, to what extent different predictors (batch ID, subject ID, age, sex etc) contribute to gene expression (Fig 1e) and identify genes with the highest absolute and relative gene expression in relevant tissues (Fig 1f).

Analyses are performed across eight cancers – for which there is a strongly implicated tissue for each – as well as Schizophrenia (SCZ), Inflammatory Bowel Disease (IBD), Alzheimer’s Disease (AD), Coronary Artery Disease (CAD), Bipolar Disorder (BD), Type 2 Diabetes (T2D), Attention-Deficit/Hyperactivity Disorder (ADHD) and Serum 25 Hydroxyvitamin D (Vitamin D). These outcomes were selected to optimise the power of the relevant GWAS and the availability of curated lists of high-confidence disease genes.

## Results

### Defining absolute and relative expression across tissues and cell types

We obtained bulk-tissue, RNA-seq gene expression data from the GTEx consortium^35^. Throughout this study, we assess two gene expression measurements: (1) absolute gene expression, representing the median number of transcripts per million (TPM) of each gene in each tissue, and (2) relative gene expression, calculated by dividing the absolute gene expression (median TPM) of each gene in a tissue by the total expression of that gene across all the other tissues (Methods). The relative gene expression measure, often referred to as gene expression specificity, has been widely used to map genes to their specific tissue and cell type expressions^14,36,37^.

### Exploring the expression profiles of disease-associated genes

To identify where in the body disease-associated genes operate, here we leverage GWAS and gene expression data utilising three alternative strategies: (i) *‘GWAS to Gene Expression’*; (ii) *‘Gene Expression to GWAS’*; and (iii) *‘Systematic Literature Search’*.

### GWAS to gene expression

Heritability across the genome is influenced by polygenicity and the genome’s correlation structure (LD), causing signals from single causal variants in key disease genes to spread across wide regions and many genes. Although the ‘omnigenic model’ suggests there may be few key (*‘core’*) disease genes despite widespread genetic associations^38^, identifying those causal variants and genes remains challenging. To link regulatory SNPs to their target genes and prioritize genes based on GWAS results, comprehensive annotations of genome function^35,39–43^ and a range of statistical and computational approaches have been developed^44–48^.

Given the absence of a single gold standard approach, we use three alternative methods to collect, for each disease, lists of putatively causal genes inferred from GWAS results: (i) *nearest-to-hit genes*: genes are prioritized by physical proximity to each GWAS hit, (ii) *fine-mapped genes:* we extracted from published studies – often produced by large GWAS consortia – lists of genes prioritized on the basis of multiple statistical and functional genomic prioritization strategies, and (iii) *PoPS genes:* derived using the recently published method Polygenic Priority Scores (PoPS)^48^, which leverages polygenic enrichment and functional gene features to prioritise genes.

#### - Nearest-to-hit genes:

While functional data can be incorporated to improve precision in assigning SNPs to genes^44^, previous research suggests that the nearest gene to the lead SNP is the most likely causal gene^44,48^. We performed clumping on the GWAS hits and identified an average of 162 ‘nearest-to-hit’ genes for each trait, with an average distance of 28Kbp between the SNP and the protein coding gene (See Methods, Table 1 and Supplemental Table 1).

#### - Fine-mapped genes:

The latest landmark GWAS of the major diseases^3–11^ have incorporated sub-studies performing gene prioritization analyses to produce lists of approximately 50–300 high-confidence causal genes for each disease. While different studies use a different selection of methods to prioritize putatively causal genes, most of them integrate GWAS results with the latest functional genomics data (Methods). Our literature search on fine-mapped genes resulted in gene lists ranging from 49 genes prioritized for Bipolar Disorder (BD) to 281 genes prioritized for Inflammatory Bowel Disorder (IBD), with an average of 126 across the 8 diseases investigated (Supplemental Table 2).

#### - PoPS genes:

The method PoPS^48^ leverages gene-level *Z*-scores from GWAS (calculated using the software MAGMA^32^), as well as gene features from single-cell gene expression data, biological pathways and predicted protein-protein interaction networks to prioritize putatively causal genes. For each trait, we extracted the genes with the top 1% PoPS scores, corresponding to 184 protein-coding genes with highest PoPS scores for each trait. The top 1% of genes threshold was selected because it provides a similar number of genes as the other two prioritization approaches, preventing biases due to differences in the number of genes included for each group of disease-associated genes (Supplemental Table 3).

**Figure 2.**
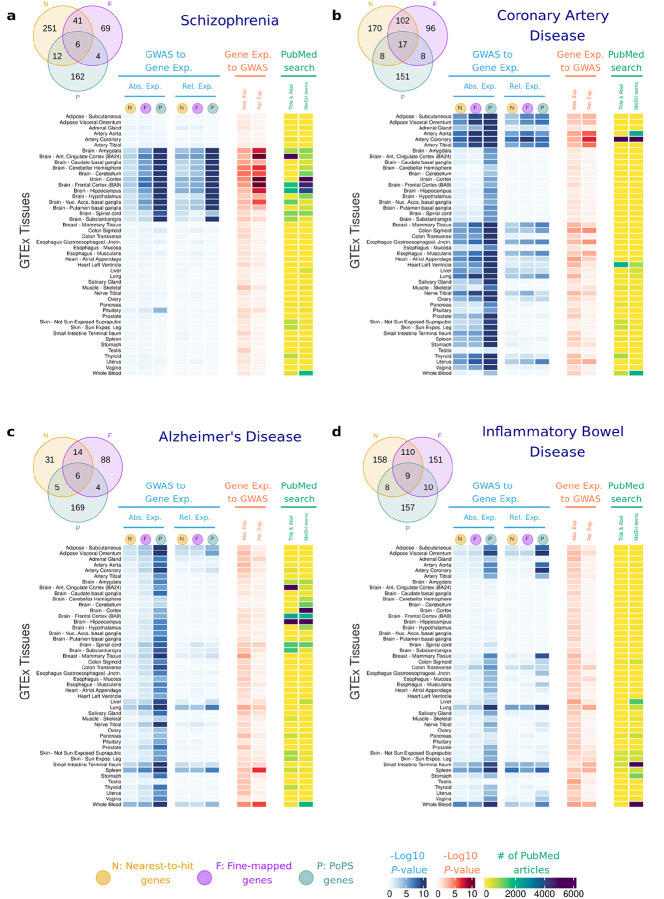

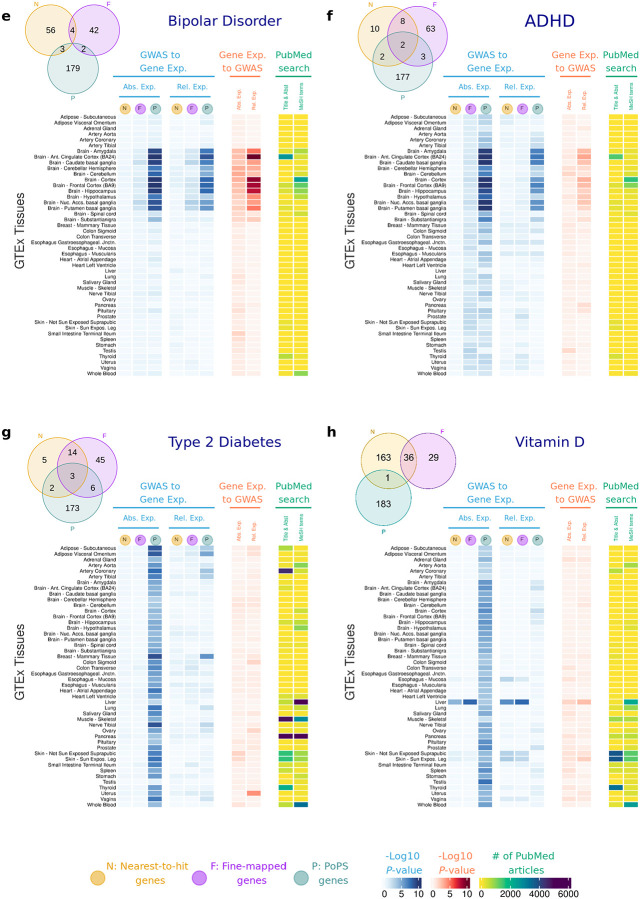
Heatmap showing results of the association between gene expression in each GTEx tissue and **a**, Schizophrenia; **b**, Coronary Artery Disease; **c**, Alzheimer’s Disease; **d**, Inflammatory Bowel Disease; **e**, Bipolar Disorder; **f**, ADHD; **g,** Type 2 Diabetes; **h**, Vitamin D. In blue, results showing the Log_10_ *P*-value for a one-side *t*-tests, testing the null hypothesis that disease-associated genes are not more expressed than other protein-coding genes expressed in that tissue. In red, results showing the Log_10_ *P*-value for enrichment of GWAS signal across the set of genes with highest absolute and relative expression for each tissue. In yellow, results for the Literature Search using PubMed. Abs. Exp, Absolute expression; Rel. Relative expression; F, Fine-mapped genes; N, Nearest-to-hit genes, P, Polygenic Priority Scores genes; Title & Abst, Title and Abstract; MeSH, MeSH terms.

The Venn diagrams in Fig 2 shows the overlap of genes for each of the diseases examined. Across the eight non-cancer diseases, fine-mapped genes and the nearest-to-hit genes showed the highest overlap, likely due to the use of genomic distance to fine-map variants and prioritize genes in the previously published studies from which we extracted the lists of fine-mapped genes. Differences in the criteria used for prioritizing genes may have partially led to differences in the number of overlapping genes. For example, PoPS scores were one of the eight strategies used for creating the list of fine-mapped genes for CAD, but PoPS scores were not used for any of the other outcomes.

For each disease, we performed *t*-tests to compare the *absolute gene expression* and *relative gene expression* between disease-associated genes (fine-mapped, nearest-to-hit and PoPS genes) vs control genes. In Fig 2, we report the t-test P-values, whereas the effect size, measured using Cohen’s D, is included in Supplemental Figure 1. The *absolute gene expression* of disease-genes vs other genes is higher in tissues with established links to each disease (Fig 2, blue columns). For instance, SCZ-associated genes are more expressed in the brain, although the *P*-values vary significantly across brain tissues (from *P*-value = 3.09×10^−2^ for the nearest gene list in substantia nigra to *P*-value = 2.01×10^−29^ for PoPS genes in the brain cortex). CAD-associated genes are more expressed in the aorta, coronary and tibial arteries (*P*-values < 10^−7^). IBD-associated genes are most expressed in the small intestine and colon transverse (*P*-values < 10^−4^ except for nearest-to-hit genes). Vitamin D genes are most expressed in the skin and in the liver, with the latter tissue being where the biologically inactive vitamin D_3_ is activated to produce 25-hydroxyvitamin D_3_^49^.

Intriguingly, other significant results point to tissues not typically linked to the disease: AD-associated genes do not show higher expression across brain tissue. Instead, the most significant differences in expression appear in the blood and spleen (PoPS genes *P*-value < 10^−13^, nearest-to-hit genes *P*-value < 10^−5^) and in adipose tissues (PoPS genes *P*-values < 10^−13^). SCZ-associated genes are more expressed in the pituitary (PoPS genes *P*-values = 3.66×10^−5^). CAD-associated genes present higher expression across multiple tissues including reproductive, adipose, and digestive systems, as well as in the lung (*P*-values < 10^−6^). IBD-associated genes present highest expression in lung (*P*-values < 10^−4^), blood and spleen (*P*-values < 10^−6^). Differences between T2D associated genes vs control genes appear most significant in breast mammary tissues (*P*-values < 10^−7^). Disease genes’ *relative gene expression t*-test results are similar to those for *absolute gene expression*, but show smaller *P*-values.

We also applied the Anderson-Darling test^50^ to assess whether disease-associated genes have a distribution of expression that differs from that of other protein-coding genes (i.e. not only in terms of mean expression). This test is more sensitive to detect differences in the tails of the distribution, in comparison to tests focusing only on mean differences (e.g. *t*-tests) or at the shape of the cumulative distribution (e.g. two-sample Kolmogorov-Smirnov)^51^. In most cases, the Anderson Darling tests reported lower *P*-values than the *t*-tests (Supplemental Figure 2). Full results for the *t*-tests and Anderson-Darling tests for each disease, each tissue, and each gene list are included in Supplemental Table 4.

Overall, genes prioritized by PoPS show smaller *P*-values across a wider range of tissues, especially for CAD, AD, T2D and Vitamin D, suggesting that PoPS prioritizes genes with higher levels of expression. In the original PoPS publication^48^, PoPS scores are combined with the genomic location to provide a list of high-confidence causal genes. However, this list has a low recall (it detects few genes for each disease). Therefore, we used the top 1% PoPS, that results in 184 genes per disease. This number of genes is similar to the GWAS fine-mapping and closest gene to locus approaches and would not lead to biases in statistical power for our tests.

One potential explanation for the differences between the disease-associated genes and all other protein coding genes is that the genes associated with any disease have distinct gene expression profiles only because they are related to human traits and diseases. Therefore, we hypothesized that if we compare our lists of disease genes with other genes that are more similar in their connection to human traits (i.e. we run t-tests comparing disease genes vs other disease genes, instead of disease genes vs any protein coding gene) the differences in gene expression levels would be attenuated. To further investigate this, we repeated our analyses using two more stringent control groups. These control groups consisted of genes associated with diseases identified through GWAS, and were extracted from the Open Targets resource^52^ (Methods). Results using Open Target control genes are consistent with analyses using all protein-coding genes. In fact, the differences in absolute and relative expression between disease-associated genes and Open Targets control genes are more significant (Supplemental Figs 3–10 and Supplemental Table 5). For example, AD-associated genes are more expressed than Open Target control genes across all tissues. For CAD, only the brain does not show a significant difference between CAD-associated genes and control genes. For SCZ, disease-associated genes show higher expression in testis and pituitary, in addition to the brain tissues. Taken together, these results demonstrate that disease genes present higher expression in particular set of tissues, even if a more restrictive criteria for the control genes group is used.

### Gene Expression to GWAS

In this section we test whether genes with high absolute and relative expression in a tissue are enriched in GWAS signal. Originally proposed by Skene et al.^53^, this approach utilizes the software MAGMA^32^ to assess the enrichment of GWAS among genes in the top decile of absolute and relative (also called specific) gene expression (see Methods). We expand the original approach here since we also investigate *absolute gene expression* – in addition to *relative gene expression* – to assess whether absolute expression may also provide valuable insights for highlighting relevant tissues.

Our findings are overall consistent with the results obtained by the *t*-tests and the Anderson-Darling tests (Fig 2, red columns). For SCZ, BP and ADHD, MAGMA results were nominally significant in numerous brain tissues (*P*-value < 0.05), such as cortex, anterior cingula, hippocampus, amygdala, or cerebellum or nucleus accumbens. The strongest results were between cortex and anterior cingula tissues and SCZ (*P*-value < 10^−12^). For CAD, numerous tissues show significant *P*-values for both absolute and relative expression, with arteries (*P*value < 10^−6^), colon sigmoid (*P*-value = 4.67×10^−6^) and esophagus (*P*-value = 8.44×10^−5^) having the strongest enrichment of GWAS signal among their most specific genes. In IBD, the intestine, blood, testis, liver, lung, and spleen are the tissues with strongest enrichment (*P*-values < 10^−3^), while AD shows the strongest result in spleen and blood (*P*-values < 10^−6^). For Vitamin D, liver is the most relevant tissue (*P*-value = 2.52×10^−3^). For T2D, none of the tissues showed significant results. Overall, MAGMA enrichments for relative gene expression are more pronounced than for absolute gene expression. MAGMA results for each disease, each tissue, and each gene list are included in Supplemental Table 6.

### Systematic Literature Search

We investigated disease-tissue associations by cross-referencing our findings with PubMed data using two methods to construct the PubMed search queries. For the first method, we use Medical Subject Headings (MeSH) terms, a standardized vocabulary from the National Library of Medicine. For the second method, we identify tissue/disease pair names in the title and abstract of the PubMed articles. Both methods provide consistent results (Fig 2, yellow columns). While the PubMed search results largely support our findings, there are additional tissues identified that may be understudied, as the number of occurrences in the literature is low. For instance, the spleen in relation to AD and IBD, as well as tissues associated with the digestive system in the context of CAD, offer promising avenues for further exploration. Results with the number of papers found for each query are included in Supplemental Table 7 & 8.

### Correlation in results across the three alternative strategies

Across the different sets of tests, we observe the strongest correlation of results between the *t*-test and Anderson-Darling tests (mean correlation across diseases and gene lists *r* = 0.729 and 42/48 correlation tests P-values < 1×10^−3^), and between *t*-tests and MAGMA (mean *r* = 0.547 and 35/48 correlation test P-values < 1×10^−3^). Correlations among the three approaches (GWAS to gene expression, gene expression to GWAS, and PubMed search) varied widely across diseases: the disease with highest correlation of results was SCZ, followed by CAD and IBD (mean correlation across tests *r* = 0.825, *r* = 0.707 and *r* = 0.691, respectively with most P-values < 1×10^−5^). Finally, the trait with the lowest correlation was T2D, (*r* = 0.269). Detailed correlation results are included in Supplemental Table 9.

### The gene expression landscape of cancer genes

Given that for a cancer we have a strong hypothesis of what is the primary tissue involved (e.g. colon for colorectal cancer), we applied the ‘nearest-to-hit’ prioritization method to cancer traits. Genetic variants associated with eight common cancers were prioritized via GWAS^54^, and the gene closest to each GWAS hit was selected (Supplemental Table 10). The number of genes found was low for most cancer traits: 12 for bladder, 13 for kidney, 16 for lung, and 28 for ovary. Colorectal (80 genes), prostate (120 genes) and breast (228 genes) were the cancers with largest number of genes associated, and significant results were observed in these three diseases with better powered GWAS: prioritized genes exhibit higher expression in prostate for prostate cancer (*P*-value = 2.94×10^−6^), in breast for breast cancer (*P*-value = 1.27×10^−4^), and gut tissues for colorectal cancer GWAS(colon *P*-value = 4.54×10^−5^, small intestine *P*-value = 7.07×10^−5^). The other cancer traits with low number of GWAS associations – and therefore lower number of genes – showed non-significant tissue-trait association results (Supplemental Figure 11 and Supplemental Table 11).

We also performed sex stratified analyses for cancers where the cancerous tissue is only available in one of the sexes (i.e. ovary, prostate, breast), and assessed the expression of disease genes across all GTEx tissues in men and women separately. Results were as expected (Supplemental Figure 12 and Supplemental Table 12): Despite the low number of genes identified in ovary cancer, the Anderson-Darling test shows a significant association for vagina in women (*P*-value = 7.62×10^−6^), and genes related to prostate cancer are highly expressed in men. In addition, the association of breast and breast cancer is less significant in men (Rel. Expression *P*-value=0.0018) than in women (Rel. Expression *P*-value=0.00012).

### Sex-stratified analyses for T2D

Since results show T2D genes in breast present higher expression than control genes, we performed sex-stratified analyses to see whether results were driven by one of the sexes. We repeated our analysis and compared the gene expression of disease *vs* control genes in men and women separately (Methods).

For T2D, disease associated genes are more expressed in breast for both sexes (Supplemental Figure 13, **panels a and b**), although *P*-values were slightly lower for men (*P*-values for Rel. and Abs. Expression < 10^−6^) than for women (*P*-values for Rel. and Abs. Expression < 10^−4^). Significant results were also observed for adipose tissues (*P*-values range: 10^−3^ to 10^−13^), pituitary in the case of women (PoPS *P*-value=0.001), and testis in the case of men (PoPS *P*-value=5.68×10^−12^). Significant results were observed only for the PoPS gene list, and had smaller *P*-values in the Anderson-Darling test than in the *t*-tests (Supplemental Table 13).

To investigate whether specific genes are driving the T2D results in a sex-specific manner, we examined the expression of individual genes for each gene list (nearest-to-hit, Fine-mapped, PoPS). Across all tissues with significant *P*-values, the gene Thymosin Beta 10 (TMSB10) is highly expressed (Supplemental Fig 13, **panel c**). TMSB10 plays an important role in the organization of the cytoskeleton by binding to acting monomers, and therefore inhibiting actin polymerization. Multiple studies have reported TMSB10 upregulation in cancer^55–58^, including pancreatic cancer^59,60^. Moreover, repositories like *MalaCards* and *Gene Cards* report the association between pancreatic cancer and TMSB10 as highly relevant. The relationship between TMSB10, T2D and pancreatic cancer is particularly interesting, given that T2D has been consistently associated with pancreatic cancer in previous epidemiological studies, with a two-fold higher risk of developing pancreatic cancer among diabetes patients^61,62^.

### Impact of highly expressed genes

To investigate whether the observed signal is primarily influenced by a small subset of genes that are highly expressed, we excluded genes within the top 10% of absolute and relative expression in *relevant tissues*, defined as the tissues where disease-associated genes were significantly more expressed than control genes (Methods). The number of tissues removed, and number of disease-associated genes are listed in Supplemental Table 14. We repeated the *t*-test and Anderson-Darling tests with these new lists of disease-associated genes. Results show that, while the *P*-values increase for all the tests, results remain consistent after removing the top 10% expressed genes (Supplemental Fig 14 and Supplemental Table 15). For example, the cortex and cingulate cortex are significantly associated with schizophrenia (Absolute expression *P*-values < 10^−20^), and the tissues most associated with AD remain being small intestine (*P*-value = 3.82×10^−9^), spleen (*P*-value = 4.91×10^−8^) and lung (*P*-value = 5.01×10^−8^).

### The gene expression landscape at the cell-type level

Tissues are composed by different cell types, each of them expressing gene expression programs to perform specific functions. The cell types in each tissue and their relative proportions may affect the results observed in the previous sections, since abundant cell types will be better powered than rare cell types for detecting differences between disease-associated genes and control genes. To further investigate where in the body disease genes operate, we repeated our testing framework in a set of cell types and tissue regions extracted from the (i) Tabula Sapiens^34^, a dataset which accrues nearly 500,000 cells from 24 different tissues and organs, many from the same donor, and (ii) ARCHS4^33^, a resource that aggregates RNA-seq data from the Gene Expression Omnibus and the Sequence Read Archive.

**Figure 3.**
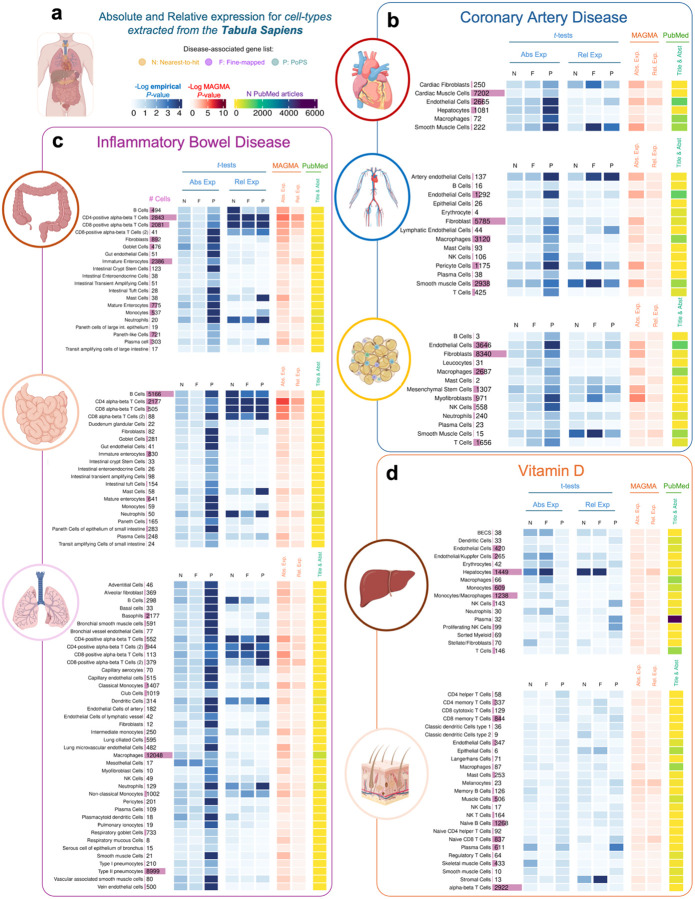
Heatmap showing results of the association between gene expression in each cell-type obtained from the Tabula Sapiens dataset **a**, Tabula Sapiens datasets were downloaded for each tissue, and absolute and relative expression was calculated for each cell type in each tissue. **b**, Results for Coronary Artery Disease. **c**, Results for Inflammatory Bowel Disease. **d**. Results for Vitamin D. In blue, results showing the Log_10_ empirical *P*-value after running 10,000 permutations, testing the null hypothesis that disease-associated genes are not more expressed than other protein-coding genes expressed in that tissue. In red, results showing the Log_10_ *P*-value for enrichment of GWAS signal across the set of genes with highest expression for each tissue. In yellow, results for the Literature Search using PubMed. Abs. Exp, Absolute expression; Rel. Exp, relative expression; F, Fine-mapped genes; N, Nearest-to-hit genes, P, Polygenic Priority Scores genes; Title & Abst, Title and Abstract are used in the PubMed Search.

**Figure 4.**
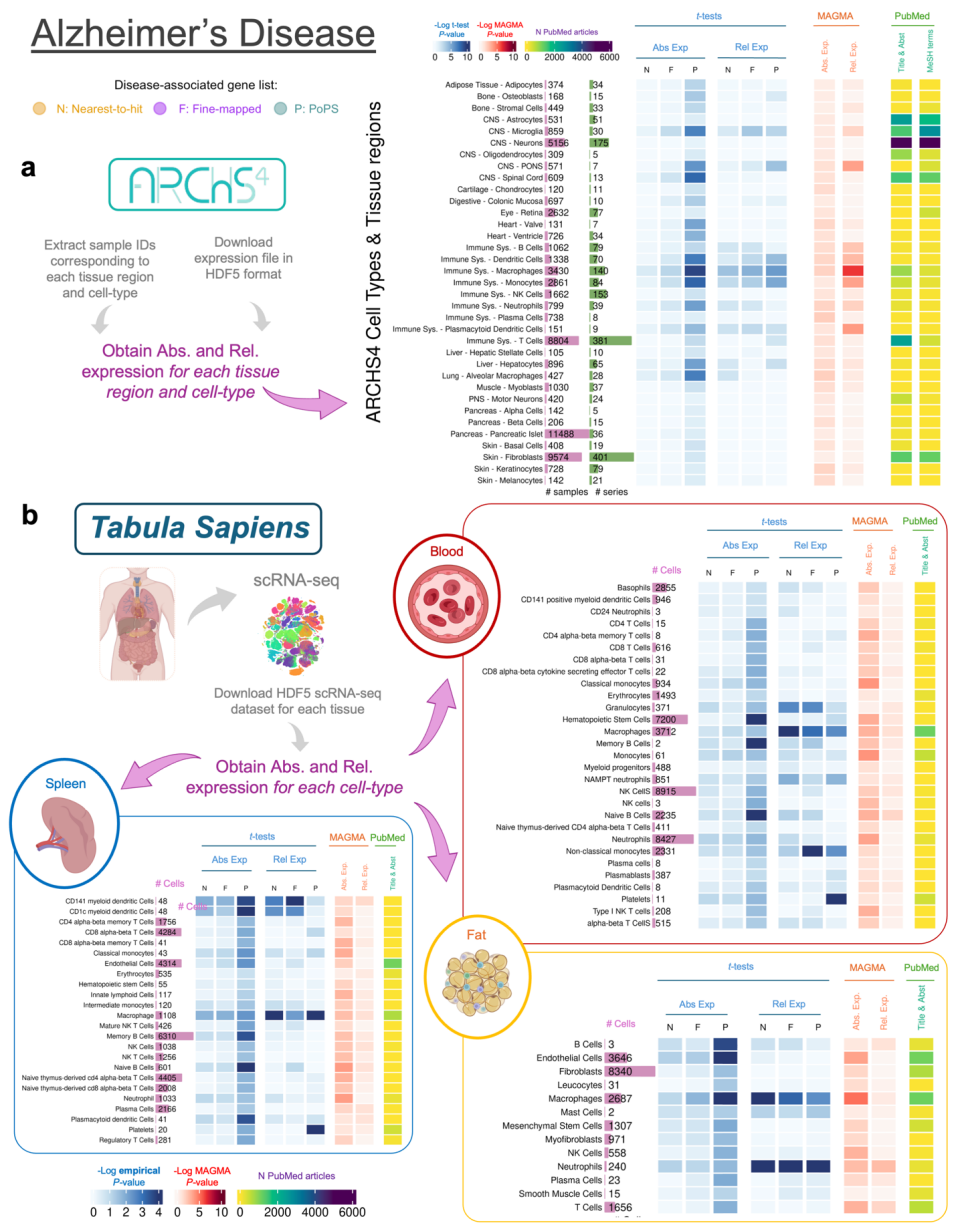
Heatmap showing results of the association between gene expression in each cell-type obtained from the Tabula Sapiens dataset. **a**, Results using RNA-seq data from cell types and tissue regions extracted from the ARCHS4 resources. **b**, Results using RNA-seq data from the Tabula Sapiens for blood (red section), spleen (blue section), and fat (yellow section).

Fig 3 presents the results of the analyses conducted with a set of tissues from Tabula Sapiens for Vitamin D, IBD and CAD, and Fig 4 presents the results of the analyses for AD using tissue regions and cell type-level datasets from both ARCHS4 (Fig 4a) and Tabula Sapiens (Fig 4b). Results for the other diseases using ARCHS4 can be found in Supplemental Fig 15 and Supplemental Tables 16–18. We focus on Vitamin D, AD and IBD because significant results were observed in “non-typical” tissues such as the spleen (primarily composed of immune cells) and lung. We also focus on CAD because this disease shows the largest number of associated tissues. Overall, the results for ARCHS4, Tabula Sapiens and GTEx are consistent, although the *P*-values for all cell-type analyses tend to be higher. In the Tabula Sapiens, PoPS genes show higher expression than control genes, but these results are often not replicated in fine-mapped genes or nearest gene lists (Supplemental Tables 19–21). Therefore, only results that replicate in at least two gene lists are reported in the following paragraphs.

Results for IBD in cell types derived from the small intestine, large intestine, and lung show that IBD-associated genes have higher expression in T-cells (*P*-values for Relative expression < 0.02). Other immune cell types such as B cells (*P*-values < 0.038), neutrophils (*P*-values < 0.034) and dendritic cells (*P*-values < 0.01) also showed significant differences, albeit with larger P-values. These findings were consistent with MAGMA results.

For CAD, the most significant differences in expression between disease-associated and control genes were observed in the relative expression of T cells in the vasculature system (*P*-value = 0.0028). Additionally, we found significant differences in relative expression in smooth muscle cells in the heart (*P*-value < 0.0271) and fat tissues (*P*-value < 0.0438 for fine-mapped genes, *P*-value < 0.0138 for PoPS and nearest genes) and cardiac fibroblasts in the heart (*P*-value < 0.045). Various immune cell types such as macrophages, mast cells, and NK cells in the vasculature system were significant (*P*-value < 0.0146) but only for PoPS genes.

For Vitamin D, the results aligned with the GTEx and ARCHS4 analyses. We observed the most significant difference in expression in the liver, specifically in hepatocytes, between disease-associated and control genes (P-values < 0.0012).

In the case of AD, Fig 4a shows results using ARCHS4, where disease-associated genes present high absolute and relative expression in immune-related cell types (e.g. Absolute and relative expression *P*-values < 0.05 for dendritic cells, macrophages and neutrophils). Fig 4b shows results using Tabula Sapiens, where similar patterns emerge as in ARCHS4. For instance, macrophages (in the spleen, blood, and fat) and neutrophils (in fat) show significant differences between disease-associated and control genes (*P*-values < 0.03). Since brain tissues were not available in Tabula Sapiens, we could only look at brain-related cell types in the ARCHS4 dataset (Supplemental Fig 15). Microglia – known to play a critical role in AD^63^- is the sole brain tissue significantly associated (*P*-value = 9.72×10^−3^ for relative expression). Genes linked to SCZ, BP, and ADHD show significant absolute and relative expression in motor neurons in ARCHS4 (*P*-value = 0.00021 for PoPS genes; *P*-value = 0.036 for fine-mapped genes; *P*-value > 0.05 for nearest-to-hit genes), but not in the broader category of neurons (even though the sample size and number of studies is larger for this cell type).

Tabula Sapiens and ARCHS4 results offer insights not easily discerned at the tissue level. For example, the results that we observe between AD and IBD in spleen, blood and lung are probably driven by the high fraction of macrophages and other innate immune system cells present within those tissues. However, both datasets have their own limitations: In the case of ARCHS4, the representation of cell types and tissue regions is less systematic than in GTEx: after quality control (Methods), 8 immune and 6 CNS-related cell types are included, with only one related to the digestive system. Furthermore, ARCHS4’s heterogeneity may have reduced power to detect associations in other diseases despite correction of batch effects. While *Tabula Sapiens*^34^ may provide a more systematic multiorgan dataset at the cellular level, the scale of this dataset is smaller (they measured single-cell RNA-seq data for a total of 15 individuals, respectively, in contrast to e.g. GTEx, which assessed more than 700 individuals). Moreover, some tissues of interest were not available here, such as brain tissues to interrogate cell types related to AD, SCZ, BP or ADHD.

### Predictors of gene expression of disease genes

In the previous sections, we demonstrated that disease-associated genes exhibit both high absolute and high relative expression in certain tissues and cell types. Although previous studies have shown that tissue type is an important predictor in gene expression^64^, in this section we expand this work by evaluating multiple factors that may contribute to gene expression variability in disease-associated genes specifically. Additionally, we assess whether the relative contributions of gene expression predictors differ significantly between disease-associated and control genes.

**Figure 5.**
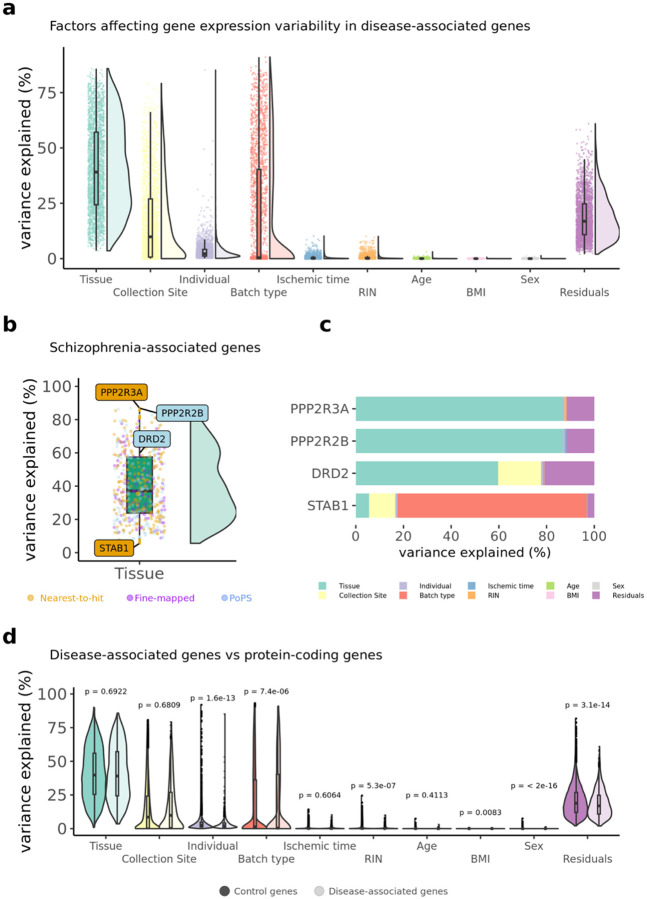
Variance partition is used to investigate the factors influencing gene expression of disease-associated genes. **a,** Violin plots representing the distribution of variance partition across all disease-associated genes for the eight diseases investigated. **b,** variance partition results for genes associated with schizophrenia. Genes labelled represent: two genes encoding protein phosphatases (PPP2R3A & PPP2R2B) where tissue-type explain a large fraction in gene expression variance, and a gene (STAB1) where tissue explains less than 10% in gene expression variance. The dopamine receptor 2 (DRD2) is also included because it is the main receptor for most antipsychotic drugs^65,66^. **c,** Bar plots of individual genes showing the variance partition estimates at the individual gene level for genes highlighted in panel b, **d,** Violin plots showing the differences in variance partition results between disease-associated genes and control genes.

To characterize the biological factors (such as tissue, sample ID, subject ID) and technical factors (such as batch ID) that contribute to variability in the gene expression of each disease-associated gene, we used the ‘*variancePartition*’ R package (v.4.3)^64^. *variancePartition* uses a linear mixed model framework in which the expression values of *each gene* are the dependent variable, and distinct sources of variation – such as those driven by tissue type, individual differences, and technical effects – are the independent variables. Overall, tissue type explains the highest proportion of the variance in gene expression (Fig 5a and Supplemental Table 22). However, there is a lot of variability across individual genes; while for some genes tissue type explains more than 70% of the variability in their expression, for other genes factors such as the type of batch, collection site, explain <20% of the variance. Fig 5b and c shows SCZ fine-mapped genes as an example of such variation in variance explained. Supplemental Figs 16–18 and the R Shiny website associated with this manuscript https://juditgg.shinyapps.io/diseasegenes/ include gene-level results quantifying the contribution of each variable to the variation in expression of each gene and disease.

Given the differences in results across genes, we tested whether the *variancePartition* results for disease-associated genes are different from all other protein-coding genes. The variance explained by biological factors (e.g. tissue, individual) and technical factors (collection site, batch type) in disease-associated genes was compared vs the variance in other protein coding genes. Individuals, batch type, RNA Integrity Number (RIN), and sex exhibited small yet significant differences in contributing to gene expression variability between disease-associated and control genes. (Fig 5d). When comparisons are assessed for each gene list and disease individually, results remain consistent. Only for Vitamin D, tissue-type explains a greater proportion of the variance for disease-associated genes than for control genes (Supplemental Figs 19–21).

### Joint evaluation of absolute and relative gene expression

**Figure 6.**
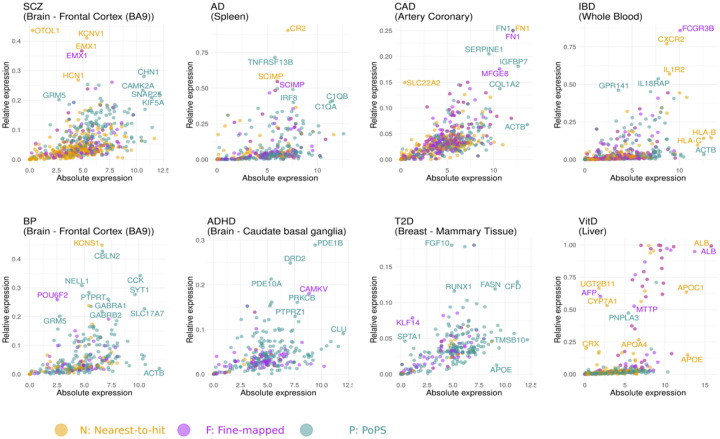
Exploring disease-associated genes showing both high absolute and high relative expression. Scatter plots showing the relationship between absolute gene expression and relative gene expression in disease-associated genes. Only the tissue with the most significant differences between disease-associated and control genes is shown.

New drugs underpinned by genetic evidence have a significantly higher success rate in clinical trials^16^. Consequently, genes prioritized via GWAS are often examined in experimental studies to validate their causal role in disease and understand their biological function. Given that (i) the *VariancePartition* results show wide variability among genes in the contribution that tissue type infers on gene expression, and (ii) the relative gene expression (a.k.a. tissue- and cell-type-specificity) of candidate target genes can inform drug efficacy^18,67^ and side effect prediction^19^, here we identify the genes with both high absolute and high relative expression across tissues. In Figure 6, we present the genes with both high absolute and relative gene expression for the tissue with the most significant differences between disease-associated and control genes. Results for the rest of tissues can be found at the R Shiny website associated with this manuscript https://juditgg.shinyapps.io/diseasegenes/.

When assessing both high absolute and relative gene expression, we find that absolute expression provides useful information beyond specificity. For example, ALB is a gene associated with vitamin D that presents high absolute and relative expression in liver, FN1 is a gene associated with CAD with high absolute and relative expression in the coronary artery. In contrast, APOE presents high expression in tissues such as breast and liver but is not specifically expressed in any of them (low relative expression), and OTOL1 is a gene associated with schizophrenia, with high relative expression in the Frontal Cortex, but low absolute expression.

Nevertheless, absolute expression alone is typically insufficient to identify the most suitable tissues for validating individual disease-associated genes because these genes often maintain elevated expression across various tissues. Supplemental Figs 22 to 29 show the co-occurrence of the 10 most expressed fine-mapped genes across diseases, showing a high overlap of highly expressed genes across multiple tissues. These results – showing that many disease genes are often expressed across many tissues – are in line with previous studies showing that 46% of protein-coding genes are expressed in all tissues^68^.

## Discussion

In this study, we have systematically characterized the gene expression features of GWAS prioritized genes. Genes associated with diseases exhibit higher absolute and relative gene expression not only in the anticipated tissues and cell types (e.g. brain in SCZ, BP and ADHD), but also in tissues and cell types not typically associated with the diseases (e.g. lung and spleen in AD and IBD, motor neurons in psychiatric disorders, cells in the PONS associated with IBD). Additional analyses removing genes with the highest expression and using more stringent criteria for the control group showed similar results. Next, we explored which biological and technical factors are significant predictors of gene expression in disease-associated genes. Although tissue-type is a consistent key contributor to gene expression variability of disease-associated genes in GTEx, results varied widely, with some disease gene showing batch and subject ID as important predictors of gene expression. Finally, and given that (i) highly expressed genes tend to maintain their elevated expression level across multiple tissues, and (ii) tissue-specific genes are reported to be twice as likely as broadly expressed genes to be drug targets^16,17^, we highlight disease genes with both absolute and relative gene expression – as these properties will be important for further experimental validation and drug target development.

We first focus our study on tissue-level analysis, despite the intense focus in the field on cell-types. We focus on tissues because: (1) extensive prior knowledge of disease-tissue associations provides a “ground truth” and thus informs the benchmarking of our approach, (2) multiple-organ, single-cell transcriptomic atlases – that systematically characterize the cell type composition of tissues – have been performed on a limited number of individuals, since they require high-coverage sequencing to obtain highly accurate single-cell expression profiles, (3) cell types present different phenotypic properties at multiple levels, which make them difficult to define and categorize^28^. Despite these challenges, we also leveraged RNA-seq data from the ARCHS4 and Tabula Sapiens resources to explore in what cell types and tissue regions GWAS signal and gene expression converges. ARCHS4 and Tabula Sapiens results highlight the gain in specificity that can be obtained when the relevant cell types are rare (e.g. microglia in the brain, where much of the GWAS signal for Alzheimer’s disease resides, but composes only ~7% of non-neuronal cells in the brain^69^). They also explain some tissue-level associations driven by the presence of relevant cell types that can be found in multiple tissues (e.g. the presence of monocytes, relevant for AD and IBD, in spleen, lung, blood etc.). However, the ARCHS4 and Tabula Sapiens analyses also highlight the challenges related to using single cell and cell-type datasets stated above. Examples of these challenges include capturing measures of gene expression for cell types with a very low number of cells that may be obtained from the same individual, and accurately defining what is a ‘cell-type’ given a gene expression program (e.g. the cell type ‘neuron’ is abundant in ARCHS4 but likely heterogenous, resulting in no significant results between neurons and psychiatric disorders).

Our approach differs from some other approaches that combine GWAS and functional genomic data to make inference disease etiology. For example, Transcriptome-Wide Association Studies (TWAS) integrate GWAS findings with expression quantitative loci (eQTLs) to investigate how genetic variations influence gene expression^47^. TWAS relies on the availability of the eQTL data and on genes with highly heritable gene expression^70^. In contrast, our strategy provides a gene expression profile of disease-associated genes regardless of eQTL data availability and gene heritability, broadening the scope for combining GWAS signal and gene expression.

Identifying disease-associated genes that are active in a wide range of tissues, including unexpected ones, is crucial because drugs often cause side effects in the tissues where their target genes are active^71^. By providing comprehensive expression profiles of disease-associated genes, we aim to support future research in validating candidate genes and developing drug targets more effectively. However, our study has several limitations. First, no gene prioritization methods are perfect, and therefore it is possible that some genes categorized as ‘disease-associated’ may not significantly contribute to disease. Additionally, the fine-mapped and PoPS prioritization approaches used functional genomics data such as tissue and cell-type specific RNA-seq, which may lead to some circularity in the analyses. Unlike previous studies^72^, the focus of this work is not to benchmark different prioritization methods, but to follow up on previously prioritized genes to assess their expression in the body; Second, our analyses were performed at the gene level, and therefore alternative mRNA transcripts were not explored here; Third, this study mainly used the GTEx dataset, considered a population control that is “normal” relative to the age of the individual and where the tissues are considered healthy. The gene expression profiles represent a healthy state and does not explore the dynamics of gene expression during disease states. However, we propose that – before studying the dynamics of gene expression between cases and controls – it is important to understand the tissues and cell types where these genes show high expression and specificity; Fourth, we observe that factors such as age, sex, and batch have minimal but varying effects on different genes. However, we did not regress out these covariates prior t-tests or AD tests: Instead, for the GTEx dataset and ARCHS4, we obtained the median TPM for each gene across all samples. For the Tabula Sapiens analyses, we calculated *P*-values based on a permutation procedure, which generates a null hypothesis drawn from the data itself but does not explicitly adjust for covariates.

In conclusion, our study on ‘the gene expression landscape of disease genes’ not only confirms established links between diseases and tissues, but also identifies unexplained tissue and cell-type-disease associations that warrant further investigation. This systematic characterization of the gene expression features of high-confidence disease genes opens new avenues for guiding experimental follow-up and drug design, ultimately advancing our understanding of disease mechanisms and response to treatment.

## Methods

### RNA-seq datasets

#### GTEx dataset

Gene expression measurements were obtained for 50 tissues from the GTEx project^35^ version 8. Median gene TPMs for each tissue were downloaded from https://gtexportal.org/home/datasets. Standard RNA-seq processing steps were applied to the dataset as follows: (1) we filtered out all non-protein-coding genes and genes not expressed in any tissue; (2) we removed the tissues with less than 100 samples, cancer or cell related tissue types (i.e. EBV-transformed lymphocytes and Leukemia cell lines); (3) we scaled the expression of each tissue such that the total is 10^6^ TPM. 45 tissues remained after quality control.

#### Sex-stratified analyses in the GTEx dataset

In tissues for which we wanted to test whether the disease-associated genes present higher absolute or relative expression in men and women specifically (i.e. T2D), we extracted the GTEx expression dataset, and for each tissue, the median gene expression of all genes was calculated for women and men separately. For each sex, we then created absolute and relative expression datasets (where columns represent tissues, and rows represent genes). To prepare sex-specific inputs for the MAGMA analyses, we generated GMT files for men and women separately, which contain the gene sets composed of the top decile of absolute and relative gene expression for each sex. T-tests, Anderson-darling tests, and MAGMA analyses were also performed separately for men and women.

#### ARCHS4 datasets

Gene expression across cell types and tissue regions were extracted from the ARCHS4^33^ resource, which provides access to uniformly processed gene counts from human RNA-seq experiments stored in the Gene Expression Omnibus (GEO) and Sequence Read Archive (SRA). Using the ARCHS4 web browser (https://maayanlab.cloud/archs4/), we systematically identified all the cell types available in the metadata search menu. For some disease-relevant cell types like microglia in AD, we entered the cell type name directly into the metadata search bar. ARCHS4 generates R scripts listing the samples related to each cell type, to facilitate their extraction from the main repository –a HDF5 file named “human_gene_v2.2.h5” available for download at https://maayanlab.cloud/archs4/download.html (Downloaded version date: 5-30-2023).

The quality control of ARCHS4 datasets and *per-gene* TPM calculation was as follows: Only cell types with more than 100 samples across all experiments available in ARCHS4 were included in our analyses. Upon obtaining the counts expression matrix for each cell type, we performed quantile normalization of samples using the function ‘*normalize.quantiles’* available on the R package (“preprocessCore”). Quantile normalization was performed on raw counts. Given that samples from a specified cell type may originate from multiple experiments with slightly different conditions, we (1) excluded experiments containing less than 10 samples, and (2) adjusted for batch effects using the package ComBat_seq^73^, which is an improved version of the popular ComBat^74^. Unlike its predecessor ComBat (designed for microarray data), ComBat_seq is tailored for RNA-Seq studies and it does not assume a normal distribution of gene expression data.

After batch correction, median TPM values for each gene were calculated in each cell type using the formula:

$$\text{TPM}=\left(\frac{\text{Number}\;\text{of}\;\text{mapped}\;\text{for}\;\text{gene}}{\text{Gene}\;\text{length}\;\text{in}\;\text{kilobases}\left(kb\right)}\right)\times 10^{6}÷\left(\text{Total}\;\text{number}\;\text{of}\;\text{mapped}\;\text{reads}\right)$$

where ‘Gene lengths in kilobases’ were calculated using the genomic coordinates indicated in a GTF file (built GRCh38), downloaded from ENSEMBL.

#### Tabula Sapiens datasets

scRNA-seq datasets were obtained from the *Tabula Sapiens* figshare (https://figshare.com/articles/dataset/Tabula_Sapiens_release_1_0/14267219). These datasets, initially in ‘.h5ad’ format, contained gene counts for each cell and metadata, and were converted into Seurat objects and then into ‘*SingleCellExperiment*’ objects to ensure compatibility with downstream analysis tools.

Data quality processing was performed: cells with zero counts across all genes were removed, and outlier cells with an extreme number of detected genes were excluded. Pseudobulk data was then generated using the *aggregateToPseudoBulk* function from the *dreamlet* R package to aggregate expression counts across cell types, according to the free cell type annotation included in the original datasets.

#### Calculating ‘Absolute gene expression ‘and ‘Relative gene expression’ values

Since the expression patterns of protein-coding genes tend to follow a negative binomial distribution, we calculated *absolute levels of gene expression* by taking the Log_2_ of the median TPM+1 values. To calculate *relative gene expression*, we divided the *absolute levels of gene expression* of each gene by its total expression across tissues. The resulting relative gene expression ranged from 0 (gene is not expressed) to 1 (gene is exclusively expressed in this tissue). The Log_2_ of the absolute and relative expression measures were used in subsequent analyses.

To calculate absolute and relative gene expression values for each gene in the *Tabula Sapiens* dataset, the total expression counts for each cell type was obtained, and normalization was performed using the *calcNormFactors* function from the *edgeR* R package. Absolute gene expression values were calculated as the total number of counts per million for each gene in each cell type (after normalization). Data was organized in data tables where each row represented a gene, and each column represented a cell type. For relative expression of a gene in a cell type, the *cellTypeSpecificity* function – which calculates the number of counts of a gene in a cell type divided by the total number of counts across cell types in that tissue – was utilized after applying the same normalization procedure as for absolute expression.

### GWAS to Gene Expression

#### Definition of disease-associated genes inferred from GWAS results.

Three different types of gene lists were inferred for each disease using GWAS results: nearest-to-hit genes, fine-mapped genes, and PoPS genes. For all the gene definitions, we obtained each gene ENSEMBL IDs using a GTF file obtained from ENSEMBL (built GRCh37.75). The list of prioritized genes for each approach and disease can be found in Supplemental Tables 1–3.

##### - Definition of ‘nearest-to-hit’ genes:

To find the genes closes to the GWAS hit, we obtained publicly available GWAS summary statistics for the diseases investigated. We performed clumping using PLINK 1.9^75^ and individual level genotype data from the UK Biobank as a reference linkage-disequilibrium (LD) panel (UK Biobank Resource under application number 18177). During clumping, variants with *P*-values ≤ 5×10^−8^ were retained, and variants within a 250 Kbp window correlated 3 0.5 with the index variant or variants with *P*-value ≥ 0.01 were removed. For each clump, the nearest protein-coding gene to the index variant was identified. We used a GTF file obtained from ENSEMBL (built GRCh37.75) to extract the gene start and gene end coordinates of each protein-coding gene. Information about the GWAS used, number of clumped variants, genes identified and distance between variant and nearest gene is included in Table 1.

##### - Definition of ‘fine-mapped genes’:

We acquired gene lists that previously fine-mapped GWAS for CAD^3^, SCZ^4^, IBD^5^, AD^79^, BD^7^, ADHD^8^, T2D^9^ and Vitamin D^80^. Most gene lists were constructed using a combination of statistical fine-mapping, transcriptome association studies, and mendelian randomization.

For CAD, the integration of eight gene prioritization predictors enabled the identification of 220 likely causal genes^3^. For SCZ, statistical fine-mapping was integrated with summary Mendelian randomization and Hi-C interaction data to obtain a list of 120 prioritized genes^4^. For IBD, we extracted the list of genes linked to variants fine-mapped, available in the Supplemental material of the study conducted by Huang and colleagues^5^. For AD, we used a review of that reported a list of genes prioritized via fine mapping of GWAS in two previous studies^10,11^. The list is available in https://github.com/sjfandrews/ADGenetics/blob/main/results/adgwas_loci.csv. For BP, fine-mapping of the GWA signals was performed and seven complementary approaches were used to prioritize 47 credible genes that were mapped to loci by at least three of the seven approaches^7^. For ADHD, fine-mapping of the most recent ADHD GWAS^8^ identified sets of credible variants for each risk locus. Credible sets were subsequently linked to genes based on genomic position, information about eQTLs, and chromatin interaction mapping in human brain tissue as implemented in FUMA. For T2D, we used a gene list containing the nearest gene of the results of a fine-mapping approach used in 380 independent association signals^9^. For Vitamin D, we extracted a list of genes published by *Manousaki* and colleagues^80^, who prioritized genes using the DEPICT method^45^ on a GWAS of serum 25 hydroxyvitamin D.

##### - Definition of ‘PoPS genes’:

The PoPS method^48^ prioritizes disease-associated genes by integrating gene-level z-scores from MAGMA^32^, single-cell gene expression data, biological pathways, and predicted protein-protein interaction networks. The original PoPS publication suggests combining PoPS scores with location information would provide a list of high-confidence genes. However, the combined PoPS+location approach leads to a low recall (it detects very few genes for each disease). Therefore, we used the top 1% PoPS, because it results in a list of 184 prioritized genes per disease. This number of genes is similar to the number of fine-mapped and nearest-to-hit genes, reducing differences in power due to the number of genes assessed. Full PoPS results can be accessed at: https://www.finucanelab.org/data.

#### Statistical analyses to compare disease-associated genes vs other genes

The *t*-test is an inferential statistic used to evaluate whether the means of two independent samples are significantly different. Here, we run one-side *t*-tests in R, testing the null hypothesis that the expression of disease-associated genes is higher than those of the control group. *t*-tests assume that the sample means are normally distributed. Since gene expression follows a negative binomial distribution, we normalized the gene expression values by taking the Log_2_ of the median TPM+1 before applying the *t*-tests.

The Anderson-Darling^50^ is a non-parametric test to evaluate whether the gene expression of disease-associated genes originates from the same distribution than the control group of genes. It tests the null hypothesis that both groups were drawn from populations with identical distributions. The Anderson-Darling test is similar to other tests assessing differences between empirical distributions (such as the two-sample Kolmogorov-Smirnov test^51^), but it is more sensitive to differences in the tails of the distribution.

For the Tabula Sapiens dataset, *P*-values to compare disease-associated genes vs control genes were calculated using a permutation approach (10,000 permutations). We calculate empirical *P*-values here to account for the small sample size of the dataset (up to 15 individuals, although typically only 2 individuals were used to extract scRNA-seq measurements for each tissue). This method avoids bias in cases where cell types are obtained from cells derived from the same individual, ensuring that results are not affected by violated assumptions of independence –which would invalidate a t-test.

### Gene Expression to GWAS

#### Definition of genes in the top decile of expression

For each tissue, we defined to gene-sets that are in the top decile of gene expression: one gene-set is composed by the 10% of genes with the highest *absolute* gene expression. The second gene-set is composed by the 10% of genes with the highest *relative* gene expression. To obtain these gene-sets, we first classified all protein-coding genes into 11 quantiles. In this classification, the 1st quantile is composed by genes without expression in a specific tissue, whereas the 11th quantile encompasses the genes with the highest expression values. We then grouped genes from the top quantile and tested their GWAS enrichment using MAGMA and the UK Biobank as a reference panel. We expanded the gene coordinates by adding a 35 kb window upstream and a 10kb window downstream of the gene. The Major Histocompatibility Complex (MHC) region was excluded from the analyses due to their long-range LD.

#### Gene set enrichment analyses with MAGMA

MAGMA^32^ is a software designed for gene-set enrichment analysis using GWAS data. It provides enrichment results at the gene-level and at the gene-set level. In gene-level analysis, MAGMA employs GWAS *P*-values to compute gene test statistics, accounting for LD structure via a reference dataset. For gene-set analysis, gene-level association stats are transformed into Z-scores, reflecting the strength of gene-phenotype associations. MAGMA uses a competitive pathway test formula: Z = β_0_ + Iβ_p_ + Cβ_k_ + ϵ where I is an indicator (1 if a gene is in pathway p, 0 if not), and C is a covariate matrix. The resulting *P*-value originates from a test on coefficient β_p_, evaluating if the phenotype shows a stronger association with genes included in the gene-set of interest versus other genes.

### Systematic Literature search

#### Search queries utilizing Medical Subject Headings (MeSH) Terminology

The Medical Subject Headings (MeSH) thesaurus is a curated collection of terms established by the National Library of Medicine. MeSH terms are valuable in recognizing content that uses different words but refers to the same concept, enhancing the accuracy and consistency of the literature search process. Leveraging MeSH terminology, we prioritized the list of 45 tissues from the GTEx dataset based on their frequency of occurrence within MeSH terms connected to scientific articles. For each pairing of tissue and disease, a search query in the format *‘<tissue name> [Mesh] AND <disease name> [Mesh]’* was used.

#### Search queries utilizing Keyword-based Literature Search

The list of tissues and cell types were ranked based on their citation frequency within the titles or abstracts of relevant scientific articles. The construction of search queries followed the format ‘*<tissue name> [Title/Abstract] AND <disease name> [Title/Abstract]’* for each unique tissue-disease pair.

#### PubMed literature Search

To determine which tissues are associated with specific diseases based on previous knowledge, we interrogated how often a combination of tissue and disease terms appeared together in published articles found on PubMed. To count the PubMed occurrences of a tissue being mentioned in relation to a disease, we used the Python library *Beautiful Soup*^81^, taking the queries defined above as input. The script performs the following tasks: it generates combinations of tissue-disease pairs, constructs search queries, sends requests to the PubMed website based on these queries, and subsequently extracts the number of search results from the webpage. The resulting count shows how frequently the tissue-disease pair appears in the body of literature. Two types of PubMed scrapping analyses we conducted based on the type of query constructed.

#### Utilizing genes associated with other diseases as control group

To generate a list of control genes associated with multiple traits and diseases, we extracted two lists of genes from the Open Targets resource^52^. The first group uses the *Open Targets ‘Gold Standards’*. The second group uses a list of genes prioritized via *Open Targets Genetics evidence,* using a machine learning method^82^ that calculates a disease-specific score to prioritize genes.

##### - Open Targets Gold standards:

This list of genes represents a repository of >400 published GWAS loci for which there is high confidence in the gene functionally implicated. The list of gold standard genes was downloaded from https://github.com/opentargets/genetics-gold-standards/blob/master/gold_standards/processed/gwas_gold_standards.191108.tsv. The final set of genes was composed by 519 protein-coding genes from 284 traits were used as gold standard control gene list. The traits with the largest number of genes were 2 diabetes (44 genes), breast carcinoma (43 genes) and prostate carcinoma (23 genes).

##### - Open Targets Genetics evidence:

This list of genes was extracted from the results of a machine-learning method used to identify the most likely causal genes^82^. This method integrates the results of 1) fine-mapping credible set analysis, 2) functional genomics data such as pathogenicity prediction, colocalization with molecular QTLs, genomic distance and chromatin interaction data to generate predictive features. The machine-learning model is supervised using the gold-standard positive GWAS loci, and a score is computed for each gene (named Locus to gene (L2G) score). The L2G score is calibrated so that a gene’s score indicates the fraction of genes at or above the score that would be expected to be true positives. Thus, we selected genes with a score >= 0.8, which assumes that 80% of the genes associated with a trait or disease in our list are causal.

Data was downloaded from the publicly available website https://platform.opentargets.org/downloads/data, section “Target - Disease evidence / Integrated list of target - disease evidence from all data sources” (version 23/09), which provides several directories with different evidence sources for the target-disease associations. However, only the ones indicating ‘genetics evidence’ were used in our analyses. From those, 3,862 protein-coding genes from 1,582 traits had L2G scores >= 0.8. The traits with the largest number of genes were height (580 genes), blood protein measurement (359 genes), and heel bone mineral density (286 genes).

#### Profiling the gene expression landscape of cancer-associated genes

We applied the *‘nearest-to-hit’* prioritization method to cancer traits by extracting genetic variants associated with eight common cancers through GWAS. These datasets are publicly available and included lists of independent, GWAS significant SNPs used to construct polygenic risk scores^54^. The SNP lists range from 22 SNPs for pancreatic cancer to 288 SNPs for breast cancer. Unlike the other diseases analysed in this study, for which we had the full summary statistics instead of only the top SNPs, we didn’t perform clumping on these SNP lists. The procedure for assigning the closest gene to each GWAS hit was the same as for *‘nearest-to-hit’* genes.

#### Definition of disease-relevant tissues for removing genes in the top decile of expression

To define the tissues that showed significant higher expression across all the tests, we defined a *P*-value threshold for association (threshold = 0.05/45×3×2, corresponding to 45 tissues, 3 lists of gene prioritization approaches, and 2 test statistics (*t*-test and Anderson-Darling test). Then, we identified the tissues for which the Anderson-Darling and the *t*-test showed *P*-values < threshold.

To test whether our association results were driven by only a few genes, we removed the genes that are in the top decile of absolute or relative expression in the relevant tissues, and repeated the ‘*GWAS to gene expression’* analyses.

#### Calculating predictors of disease-associated gene expression

To uncover the key contributors to the variability in gene expression among disease-associated genes, we performed variance partition analyses using the R package ‘*variancePartition*’^64^. This package assesses drivers of variation for each gene by fitting a linear fixed model to quantify the contribution of tissues, individuals, technical variables etc. in gene expression.

We calculated the variance partition for each disease-associated and control gene. We used as predictors uncorrelated variables (r^2^ < 0.75) that explained the largest proportion of variance in gene expression, as calculated by the Canonical Correlation Analysis in the *variancePartition* package and reported in the original *variancePartition* publication^64^ (which also used the GTEx dataset). The variance partition analysis results in a data table where each row is a gene, and each column is the predictor variable included in the model. The results show, for each gene, the percentage of variance explained for each predictor.

## Supplementary Material

Supplement 1

Supplement 2

## Figures and Tables

**Table 1.** Gene prioritization based on the nearest gene to GWAS hit. Table shows references for each GWAS summary statistics, the number of clumped SNPs, genes and median distance between SNP and selected gene.

Trait	GWAS summary statistics used	N clumped SNPs	N unique ensemble IDs	Median distance between SNP and nearest protein coding gene (bp)
CAD	Aragam et al, 2022^3^	525	297	14,734
SCZ	Trubetskoy et al. 2022^4^	452	310	18,125
IBD	Liu et al, 2015^76^	487	285	11,724
AD	Bellenguez et al, 2022^11^	239	99	8,363
BD	Mullins et al, 2021^77^	73	63	20,660
ADHD	Demontis et al, 2023^8^	32	22	104,008
T2D	Suzuki et al, 2023^78^	50	31	26,633
Vitamin D	Revez et al, 2020^6^	517	200	22,111
