# Supplementary material for: The Gene Expression Landscape of Disease Genes
Source: medRxiv. 2024 Jun 21:2024.06.20.24309121. Preprint. [Version 1] doi: 10.1101/2024.06.20.24309121 (PMC11213058; doi:10.1101/2024.06.20.24309121)
Supplement: Supplementary file 1 [file media-2.pdf]

Supplemental Material for:

The Gene Expression Landscape of Disease Genes

Judit García-González[1], Saul García González[1,2], Lathan Liou[1], Paul F. O'Reilly[1],

[1]Department of Genetics and Genomic Sciences, Icahn School of Medicine, Mount Sinai, New York City, NY 10029, USA

[2]Center for Excellence in Youth Education, Icahn School of Medicine, Mount Sinai, New York City, NY 10029, USA

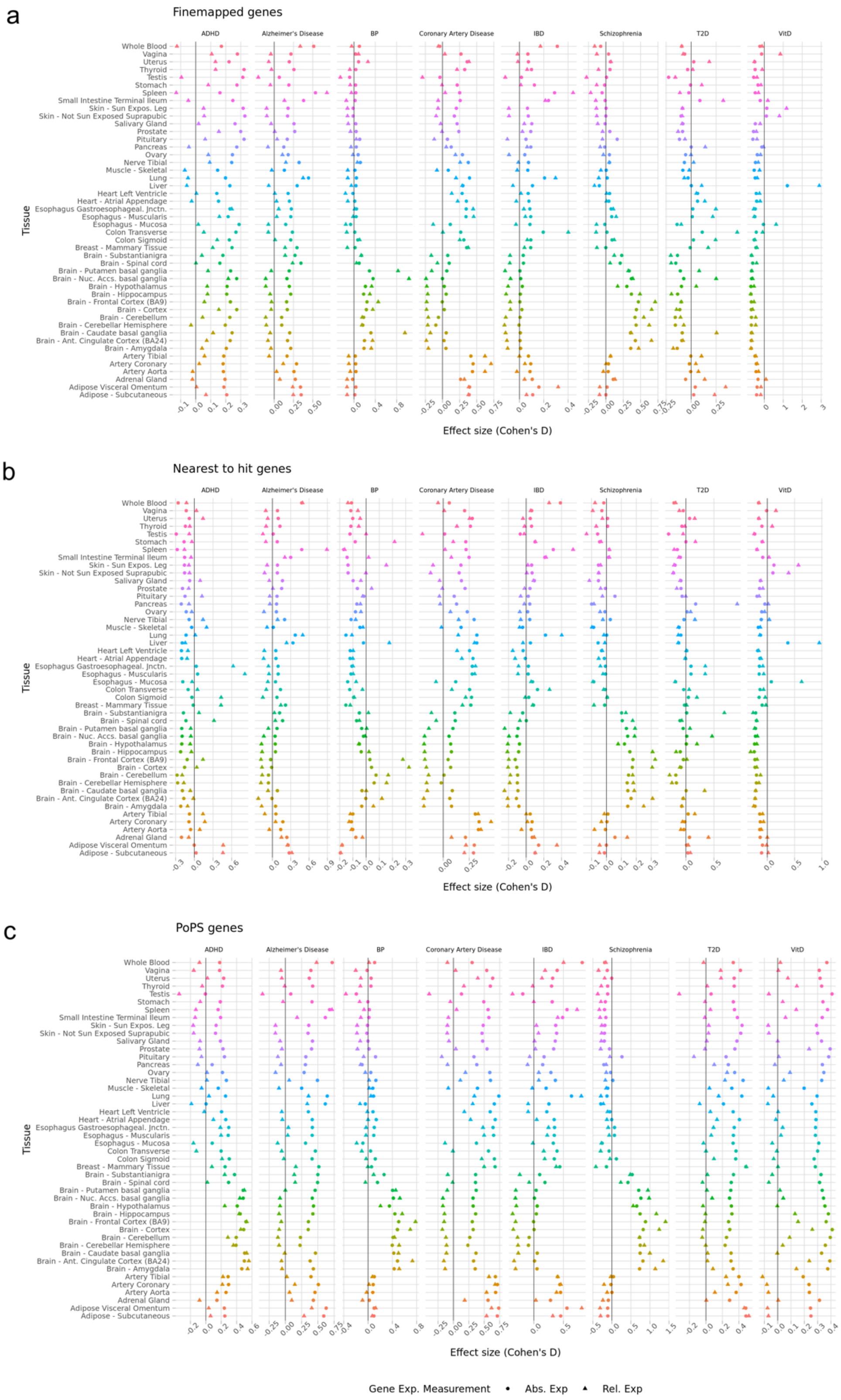

Supplemental Figure 1. Effect size (Cohen's D) across tissues for a) fine-mapped genes, b) Nearest to hit genes and c), PoPs genes. Effect sizes are shown across all the GTEx tissues, and all the diseases investigated. Absolute and relative expression are indicated as circles (absolute expression) and triangles (relative expression).

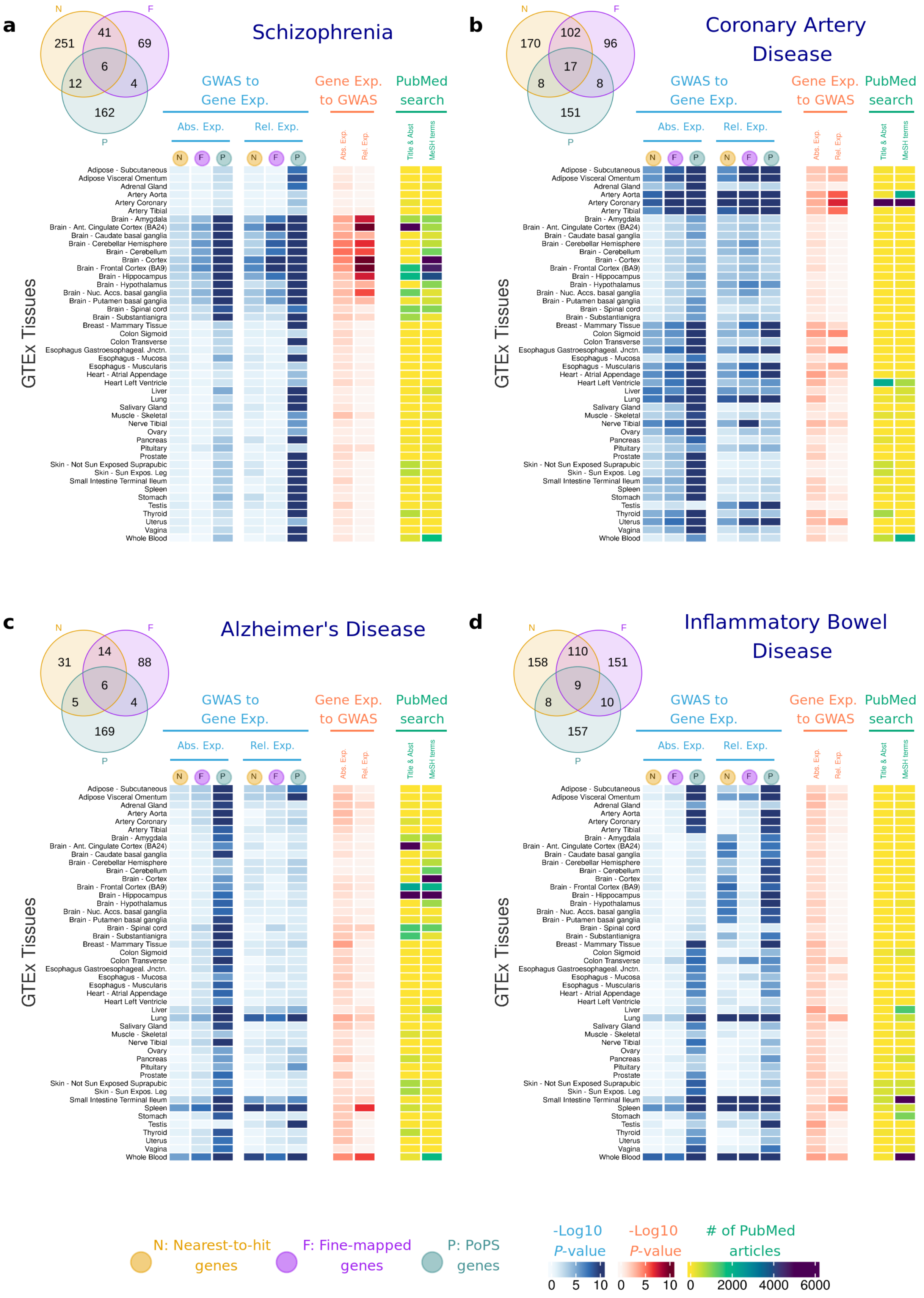
a
N
F
P
251
41
69
6
12
4
162
Schizophrenia
GWAS to Gene Exp.
Gene Exp. to GWAS
PubMed search
Abs. Exp.
Rel. Exp.
Title & Abst
MeSH terms
N F P
GTEx Tissues
Adipose - Subcutaneous
Adipose Visceral Omentum
Adrenal Gland
Artery Aorta
Artery Coronary
Artery Tibial
Brain - Amygdala
Brain - Ant. Cingulate Cortex (BA24)
Brain - Caudate basal ganglia
Brain - Cerebellar Hemisphere
Brain - Cerebellum
Brain - Cortex
Brain - Frontal Cortex (BA9)
Brain - Hippocampus
Brain - Hypothalamus
Brain - Nuc. Accs. basal ganglia
Brain - Putamen basal ganglia
Brain - Spinal cord
Brain - Substantianigra
Breast - Mammary Tissue
Colon Sigmoid
Colon Transverse
Esophagus Gastroesophageal. Jnctn.
Esophagus - Mucosa
Esophagus - Muscularis
Heart - Atrial Appendage
Heart Left Ventricle
Liver
Lung
Salivary Gland
Muscle - Skeletal
Nerve Tibial
Ovary
Pancreas
Pituitary
Prostate
Skin - Not Sun Exposed Suprapubic
Skin - Sun Expos. Leg
Small Intestine Terminal Ileum
Spleen
Stomach
Testis
Thyroid
Uterus
Vagina
Whole Blood
b
170
102
96
17
8
8
151
Coronary Artery Disease
c
31
14
88
6
5
4
169
Alzheimer's Disease
d
158
110
151
9
8
10
157
Inflammatory Bowel Disease
N: Nearest-to-hit genes
F: Fine-mapped genes
P: PoPS genes
-Log10 P-value
-Log10 P-value
# of PubMed articles
0 5 10
0 5 10
0 2000 4000 6000

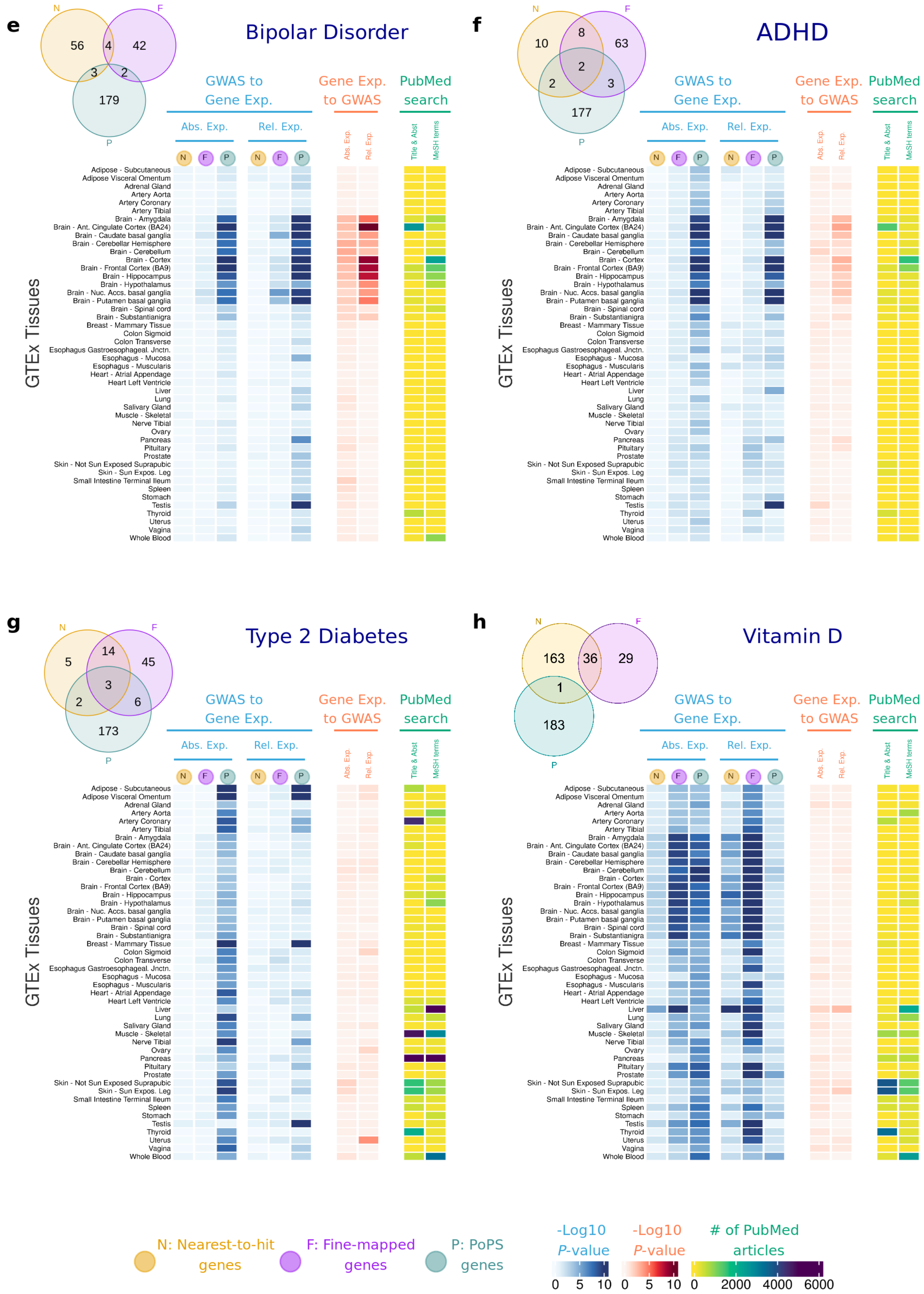
e
Bipolar Disorder
N
F
P
56
4
42
3
2
179
f
ADHD
10
8
63
2
2
3
177
g
Type 2 Diabetes
5
14
45
3
2
6
173
h
Vitamin D
163
36
29
1
183
GWAS to Gene Exp.
Gene Exp. to GWAS
PubMed search
Abs. Exp.
Rel. Exp.
Title & Abst
MeSH terms
GTEx Tissues
Adipose - Subcutaneous
Adipose Visceral Omentum
Adrenal Gland
Artery Aorta
Artery Coronary
Artery Tibial
Brain - Amygdala
Brain - Ant. Cingulate Cortex (BA24)
Brain - Caudate basal ganglia
Brain - Cerebellar Hemisphere
Brain - Cerebellum
Brain - Cortex
Brain - Frontal Cortex (BA9)
Brain - Hippocampus
Brain - Hypothalamus
Brain - Nuc. Accs. basal ganglia
Brain - Putamen basal ganglia
Brain - Spinal cord
Brain - Substantianigra
Breast - Mammary Tissue
Colon Sigmoid
Colon Transverse
Esophagus Gastroesophageal. Jnctn.
Esophagus - Mucosa
Esophagus - Muscularis
Heart - Atrial Appendage
Heart Left Ventricle
Liver
Lung
Salivary Gland
Muscle - Skeletal
Nerve Tibial
Ovary
Pancreas
Pituitary
Prostate
Skin - Not Sun Exposed Suprapubic
Skin - Sun Expos. Leg
Small Intestine Terminal Ileum
Spleen
Stomach
Testis
Thyroid
Uterus
Vagina
Whole Blood
N: Nearest-to-hit genes
F: Fine-mapped genes
P: PoPS genes
-Log10 P-value
0 5 10
-Log10 P-value
0 5 10
# of PubMed articles
0 2000 4000 6000

Supplemental Figure 2: Heatmap showing results of the association between gene expression in each GTEx tissue and **a**, Schizophrenia; **b**, Alzheimer’s Disease; **c**, Coronary Artery Disease; **d**, Inflammatory Bowel Disease; **e**, Bipolar Disorder; **f**, ADHD; **g,** Type 2 Diabetes; **h**, Vitamin D. In blue, results showing the P-value of the **Anderson-Darling test**, (as opposed to the One side T-test shown in Main Figure 2). In red, results showing the P-value for enrichment of GWAS signal across the set of genes with highest and most specific expression for each tissue. In yellow, results for the Literature Search using PubMed. F, Fine-mapped genes; N, Nearest-to-hit genes, P, Polygenic Priority Scores genes; TiAb, Title and Abstract are used in the PubMed Search; MeSH, MeSH terms are used in the PubMed Search.

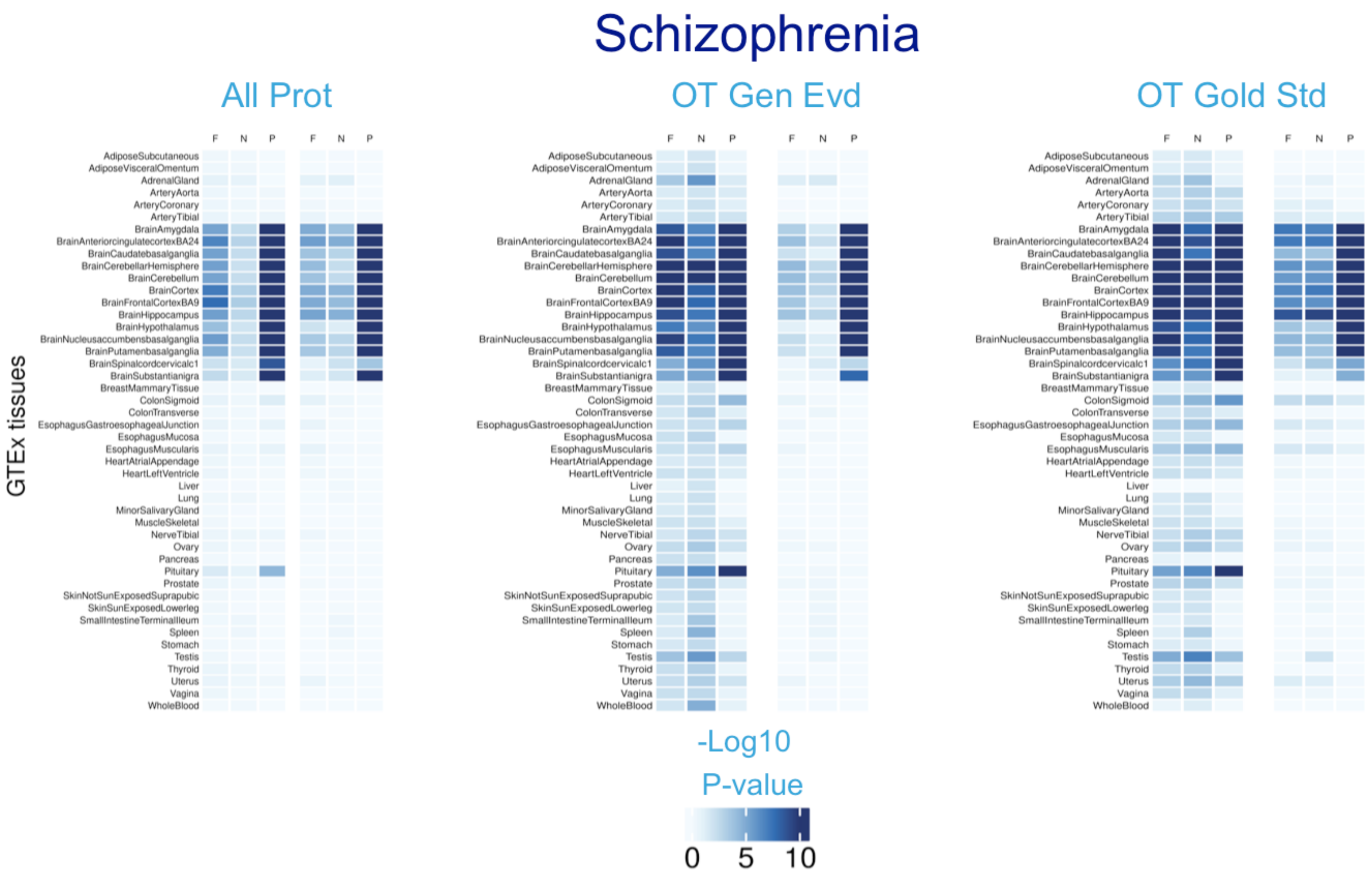

Supplemental Figure 3: Heatmaps showing T-test results of the association between gene expression in each GTEx tissue for Schizophrenia. For each disease, three panels are shown showing <u>three different control groups</u> used in the T-test: (i) *"All Prot":* all protein-coding genes, (ii) *"OT Gen Evd":* genes prioritized via Open Targets Genetics evidence and (iii) *"OT Gold Std":* Open Targets gold-standard evidence genes. Within each panel, the three first columns represent results for *Absolute* gene expression, and the second three columns represent *Specific* gene expression. F: Fine-mapped genes, N: Nearest-to-hit genes, P: PoPS genes.

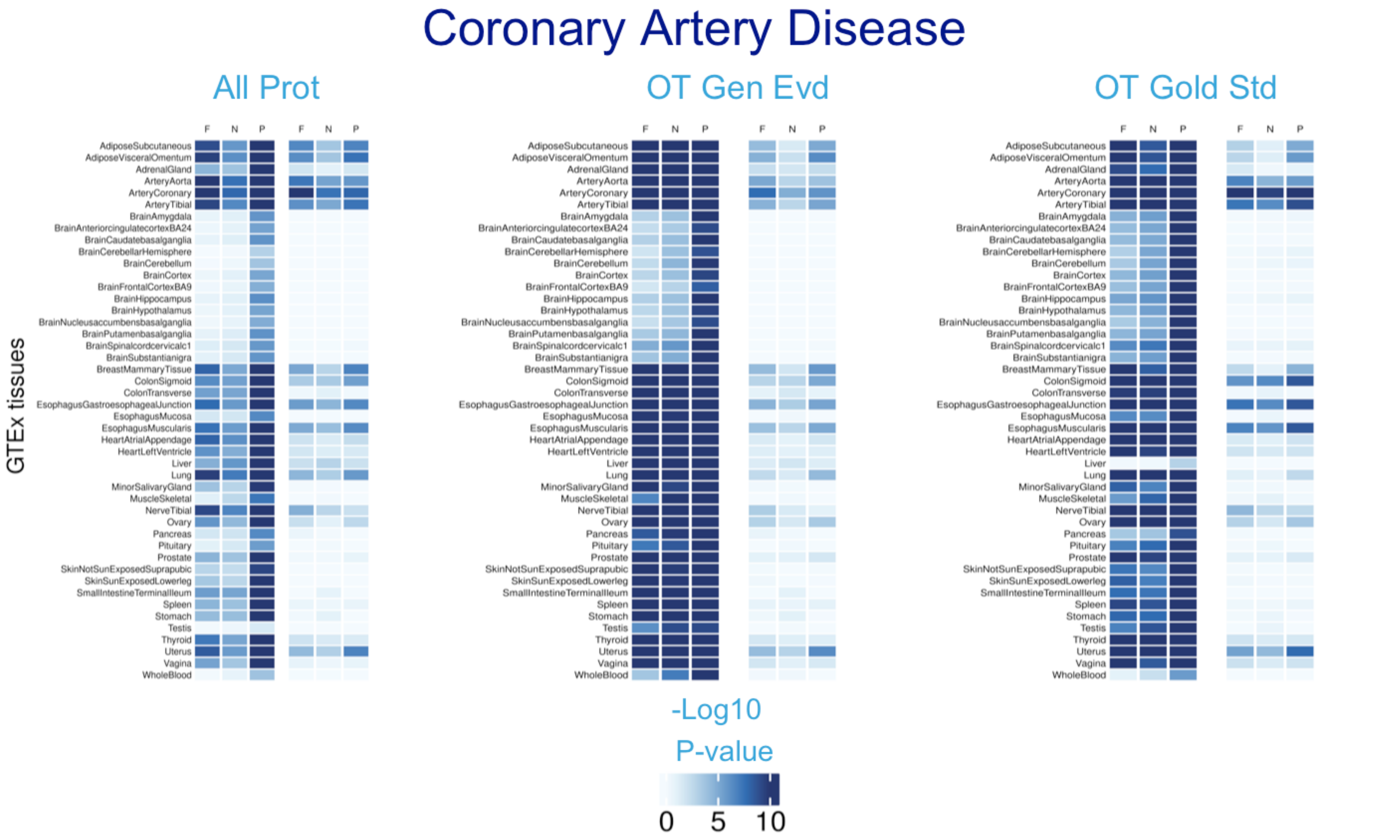

Supplemental Figure 4: Heatmaps showing T-test results of the association between gene expression in each GTEx tissue for Coronary Artery Disease. For each disease, three panels are shown showing three different control groups used in the T-test: (i) *"All Prot"*: all protein-coding genes, (ii) *"OT Gen Evd"*: genes prioritized via Open Targets Genetics evidence and (iii) *"OT Gold Std"*: Open Targets gold-standard evidence genes. Within each panel, the three first columns represent results for *Absolute* gene expression, and the second three columns represent *Specific* gene expression. F: Fine-mapped genes, N: Nearest-to-hit genes, P: PoPS genes.

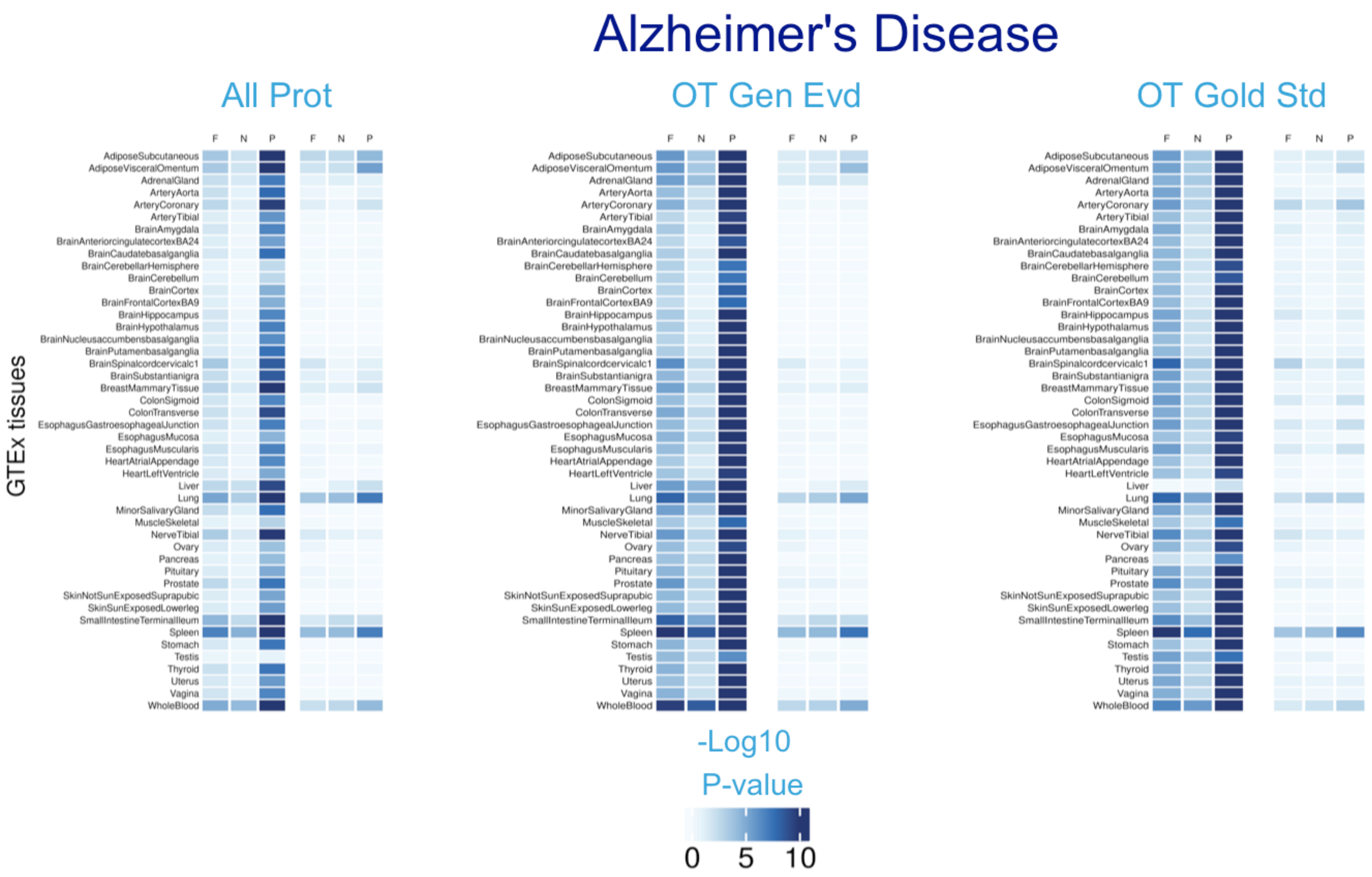

Supplemental Figure 5: Heatmaps showing T-test results of the association between gene expression in each GTEx tissue for Alzheimer's Disease. For each disease, three panels are shown showing three different control groups used in the T-test: (i) *"All Prot"*: all protein-coding genes, (ii) *"OT Gen Evd"*: genes prioritized via Open Targets Genetics evidence and (iii) *"OT Gold Std"*: Open Targets gold-standard evidence genes. Within each panel, the three first columns represent results for *Absolute* gene expression, and the second three columns represent *Specific* gene expression. F: Fine-mapped genes, N: Nearest-to-hit genes, P: PoPS genes.

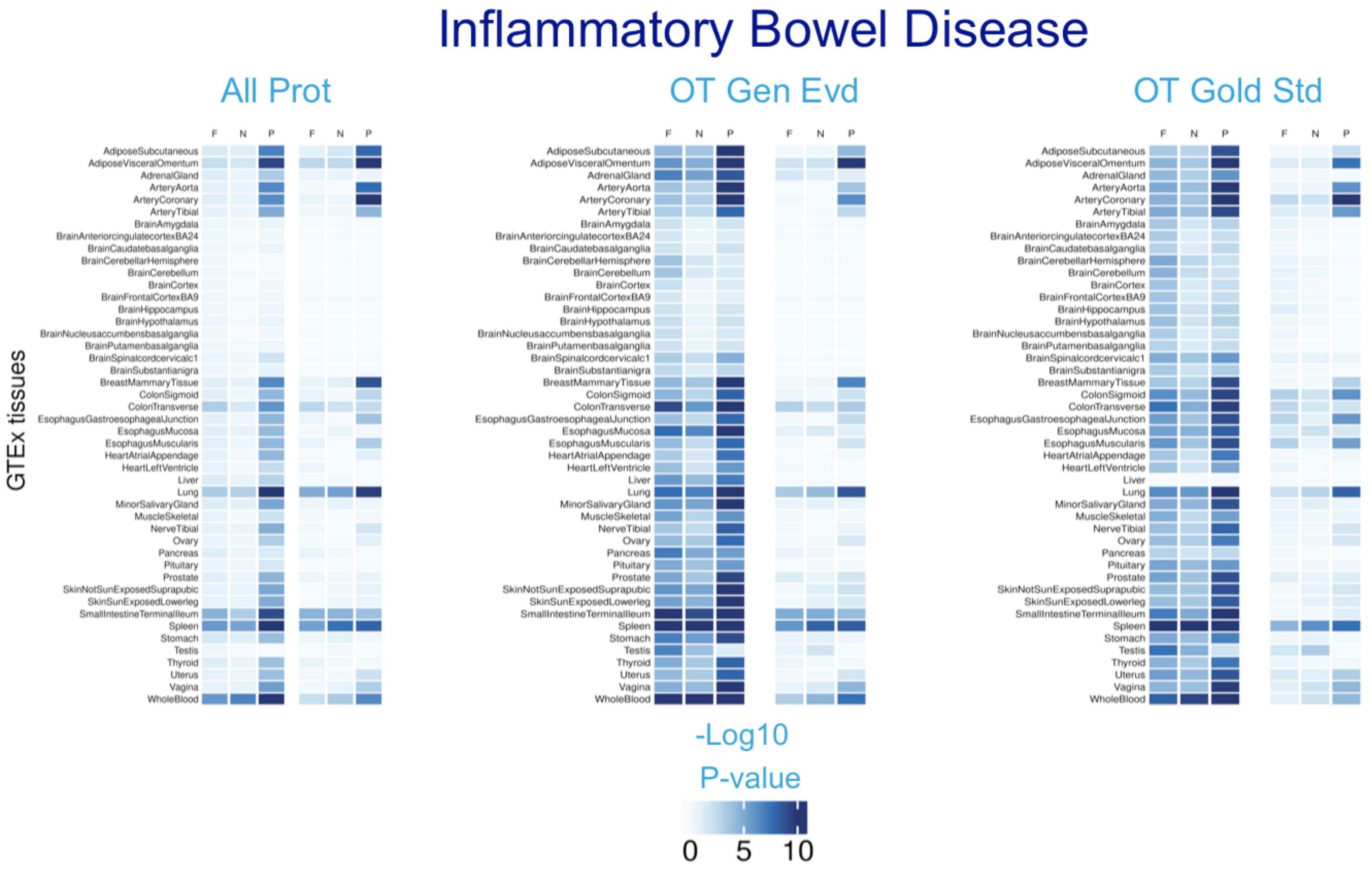

Supplemental Figure 6: Heatmaps showing T-test results of the association between gene expression in each GTEx tissue for Inflammatory Bowel Disease. For each disease, three panels are shown showing three different control groups used in the T-test: (i) *"All Prot":* all protein-coding genes, (ii) *"OT Gen Evd":* genes prioritized via Open Targets Genetics evidence and (iii) *"OT Gold Std":* Open Targets gold-standard evidence genes. Within each panel, the three first columns represent results for *Absolute* gene expression, and the second three columns represent *Specific* gene expression. F: Fine-mapped genes, N: Nearest-to-hit genes, P: PoPS genes.

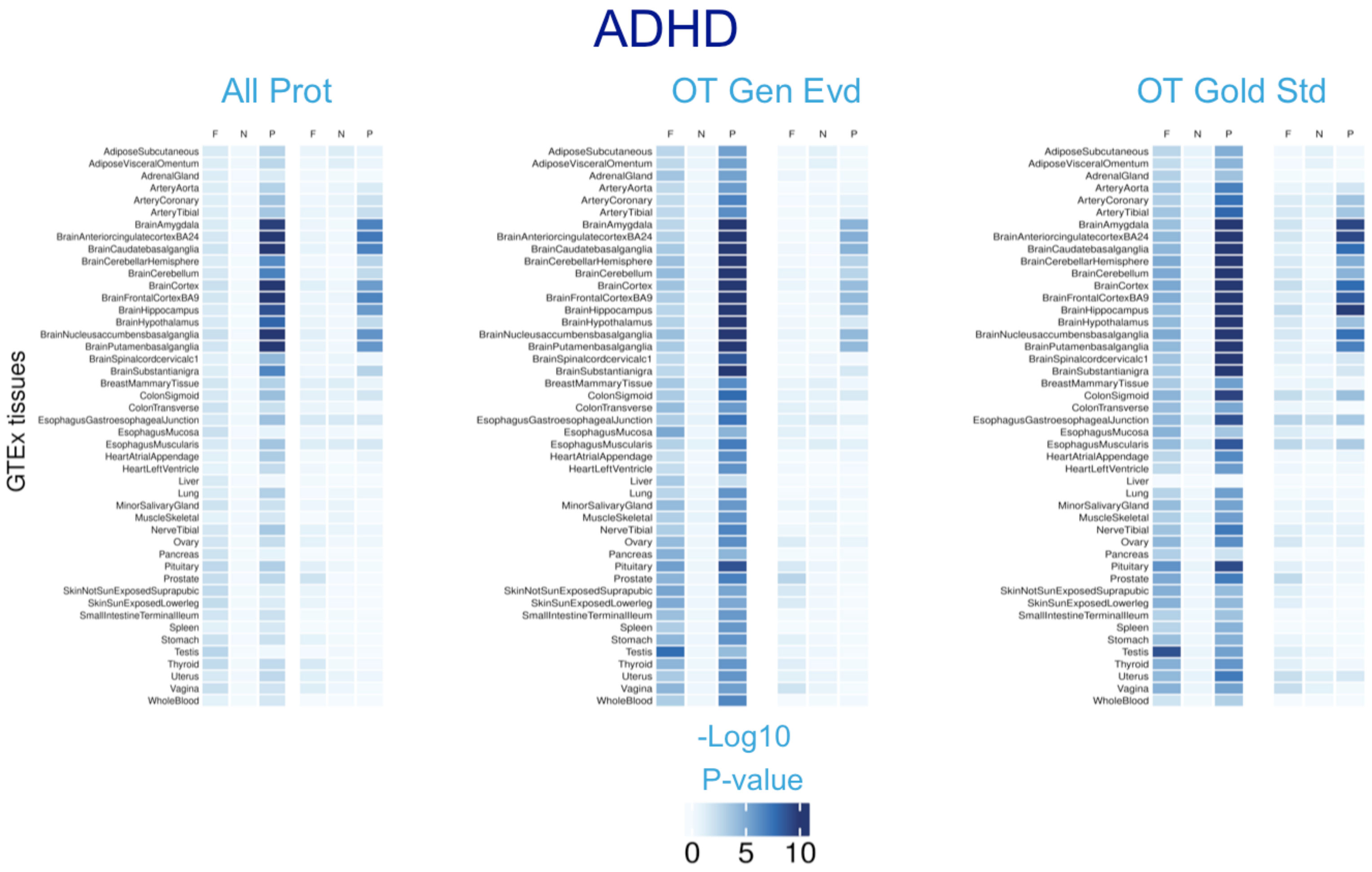

Supplemental Figure 7: Heatmaps showing T-test results of the association between gene expression in each GTEx tissue for ADHD. For each disease, three panels are shown showing three different control groups used in the T-test: (i) *"All Prot"*: all protein-coding genes, (ii) *"OT Gen Evd"*: genes prioritized via Open Targets Genetics evidence and (iii) *"OT Gold Std"*: Open Targets gold-standard evidence genes. Within each panel, the three first columns represent results for *Absolute* gene expression, and the second three columns represent *Specific* gene expression. F: Fine-mapped genes, N: Nearest-to-hit genes, P: PoPS genes.

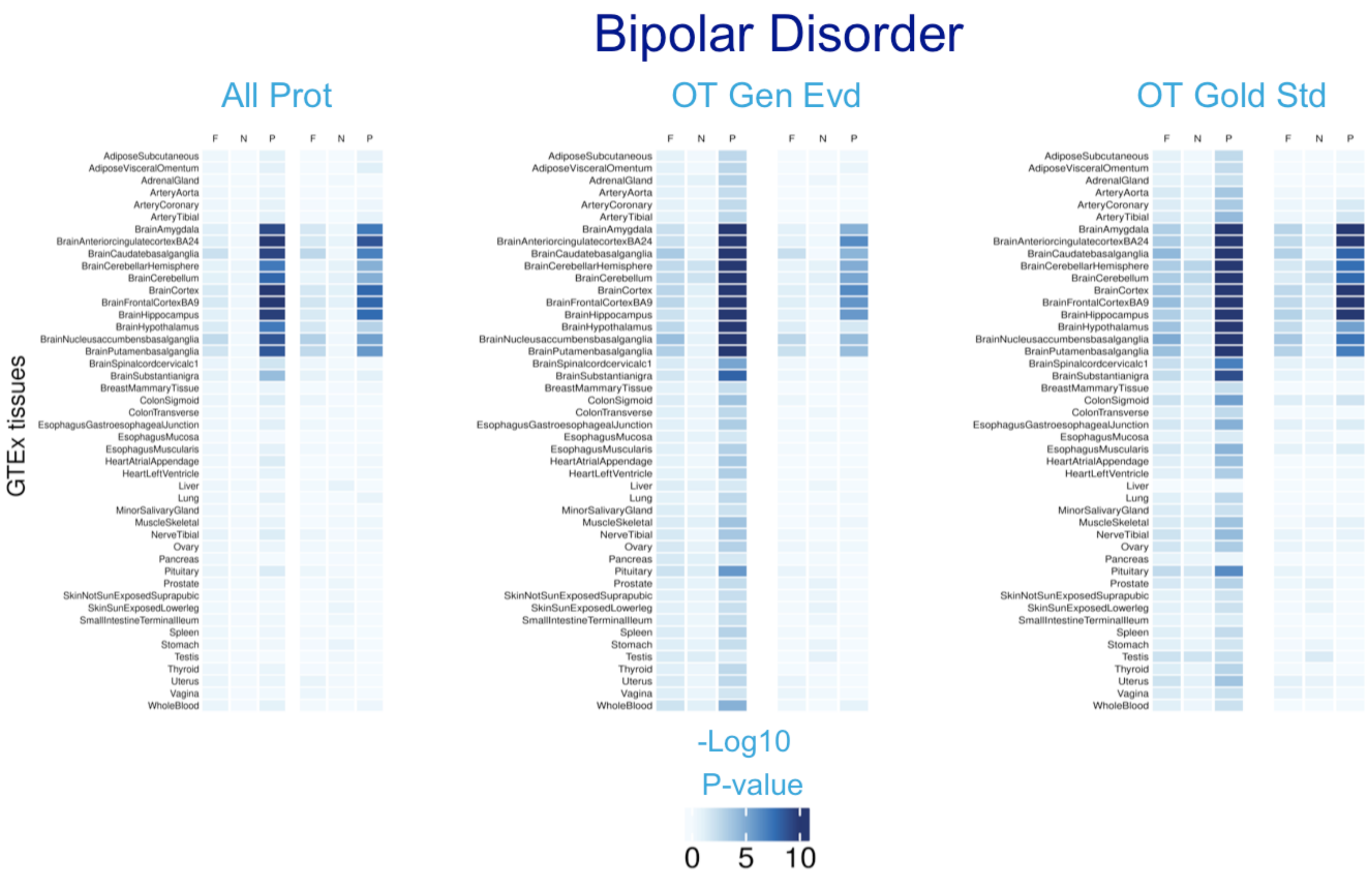

Supplemental Figure 8: Heatmaps showing T-test results of the association between gene expression in each GTEx tissue for Bipolar Disorder. For each disease, three panels are shown showing <u>three different control groups</u> used in the T-test: (i) *"All Prot"*: all protein-coding genes, (ii) *"OT Gen Evd"*: genes prioritized via Open Targets Genetics evidence and (iii) *"OT Gold Std"*: Open Targets gold-standard evidence genes. Within each panel, the three first columns represent results for *Absolute* gene expression, and the second three columns represent *Specific* gene expression. F: Fine-mapped genes, N: Nearest-to-hit genes, P: PoPS genes.

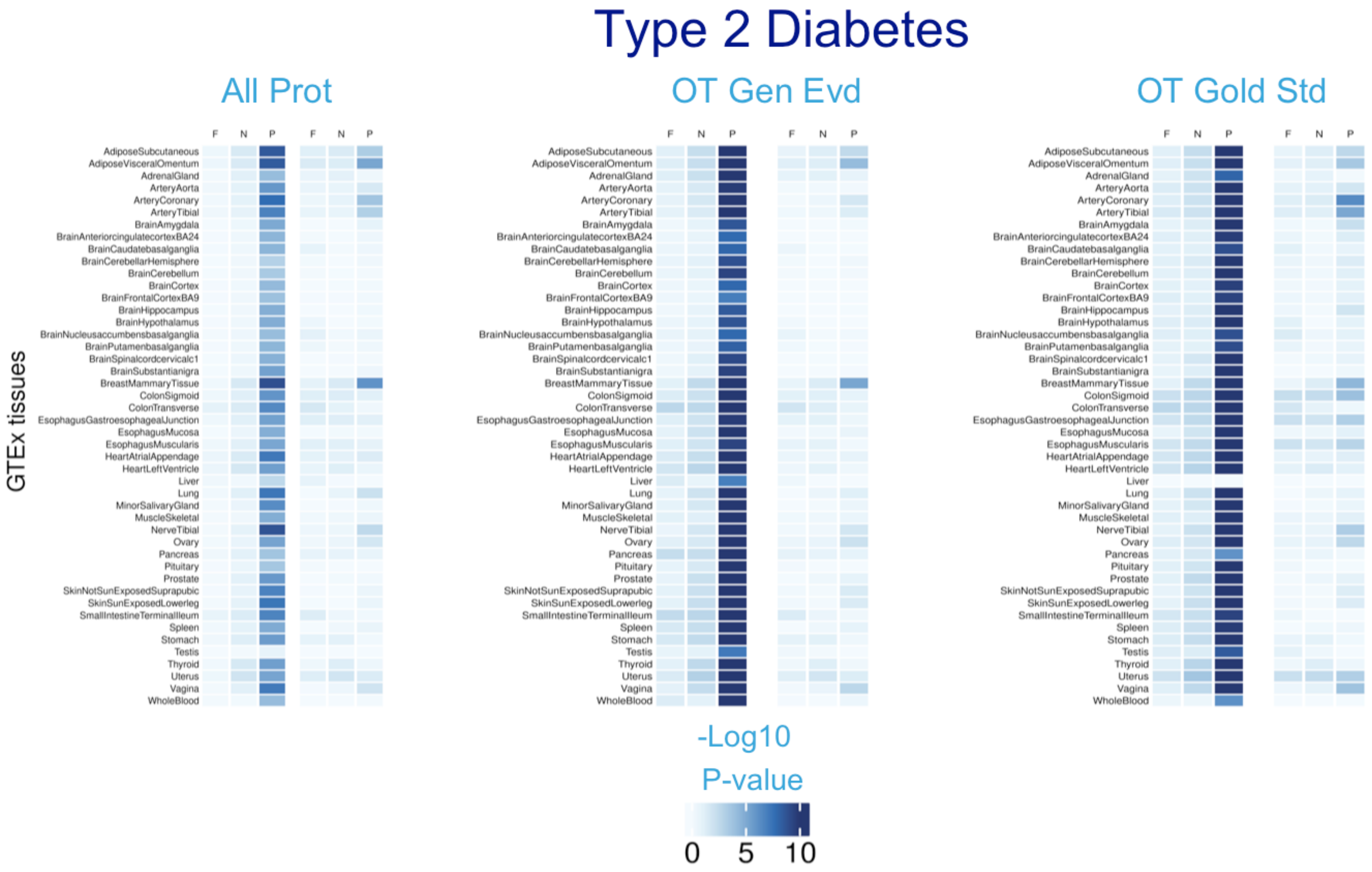

Supplemental Figure 9: Heatmaps showing T-test results of the association between gene expression in each GTEx tissue for Type 2 Diabetes. For each disease, three panels are shown showing three different control groups used in the T-test: (i) *"All Prot":* all protein-coding genes, (ii) *"OT Gen Evd":* genes prioritized via Open Targets Genetics evidence and (iii) *"OT Gold Std":* Open Targets gold-standard evidence genes. Within each panel, the three first columns represent results for *Absolute* gene expression, and the second three columns represent *Specific* gene expression. F: Fine-mapped genes, N: Nearest-to-hit genes, P: PoPS genes.

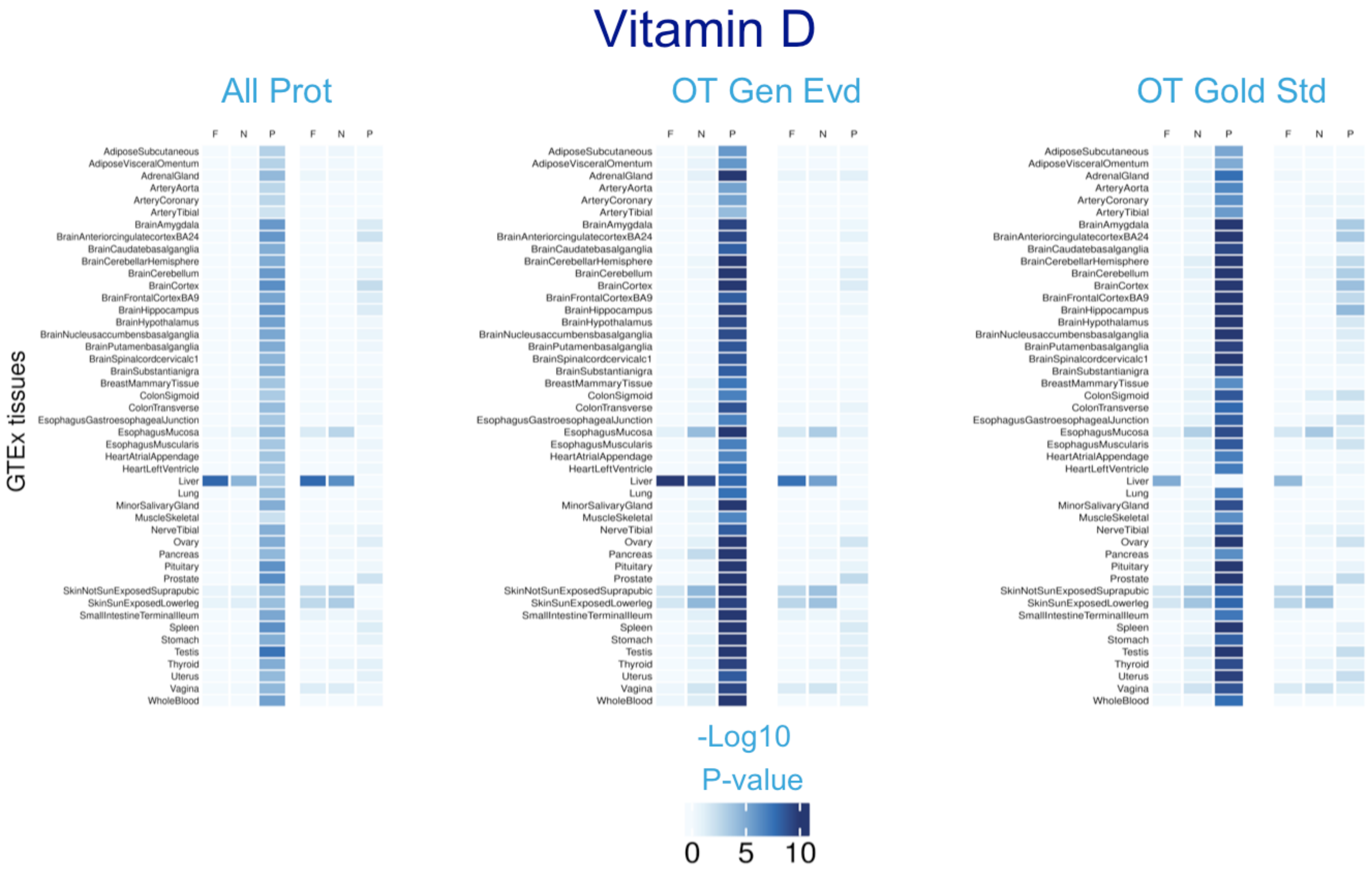

Supplemental Figure 10: Heatmaps showing T-test results of the association between gene expression in each GTEx tissue for Vitamin D. For each disease, three panels are shown showing three different control groups used in the T-test: (i) *"All Prot"*: all protein-coding genes, (ii) *"OT Gen Evd"*: genes prioritized via Open Targets Genetics evidence and (iii) *"OT Gold Std"*: Open Targets gold-standard evidence genes. Within each panel, the three first columns represent results for *Absolute* gene expression, and the second three columns represent *Specific* gene expression. F: Fine-mapped genes, N: Nearest-to-hit genes, P: PoPS genes.

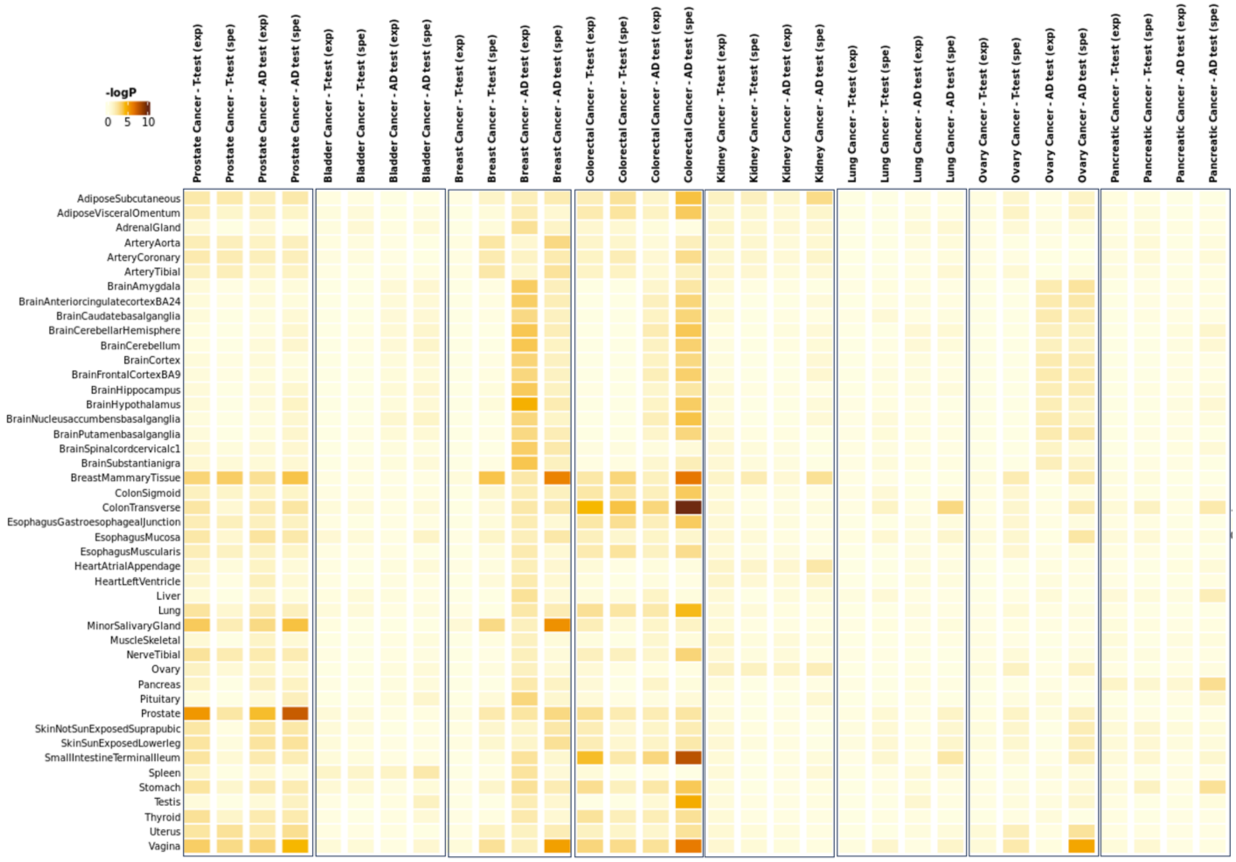

Supplemental Figure 11: Heatmaps showing T-tests and Anderson-Darling (AD test) results of the association between gene expression in each GTEx tissue. exp, Absolute gene expression was measured; spe, Specific (relative) gene expression was measured; AD, Anderson-Darling test.

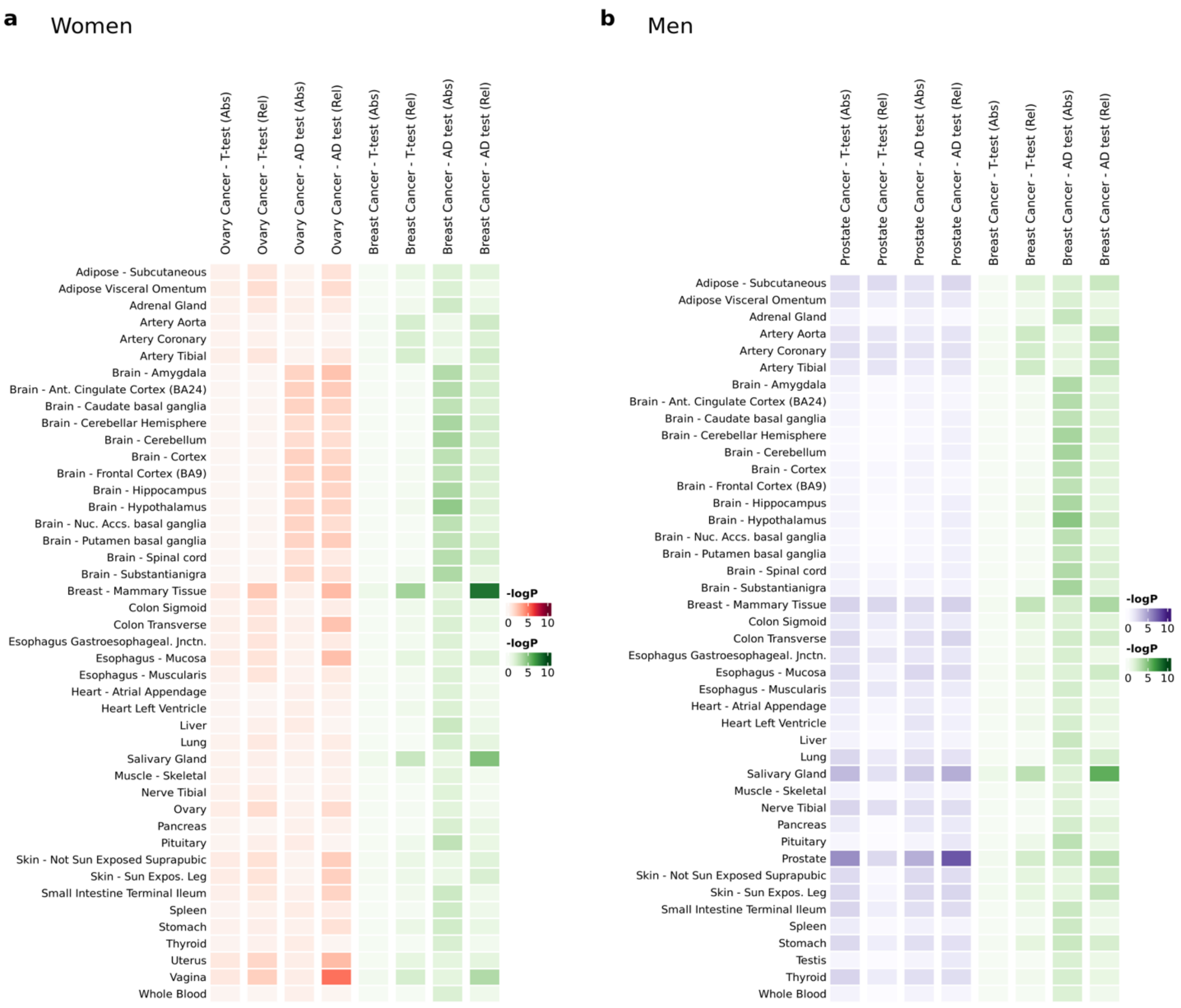

Supplemental Figure 12: Sex-stratified analyses for three cancer traits: ovary cancer, breast cancer, and prostate cancer. Heatmaps show *P*-values for the T-tests and Anderson-Darling (AD test) results of the association between absolute (Abs) and Relative (Rel) gene expression in each GTEx tissue.

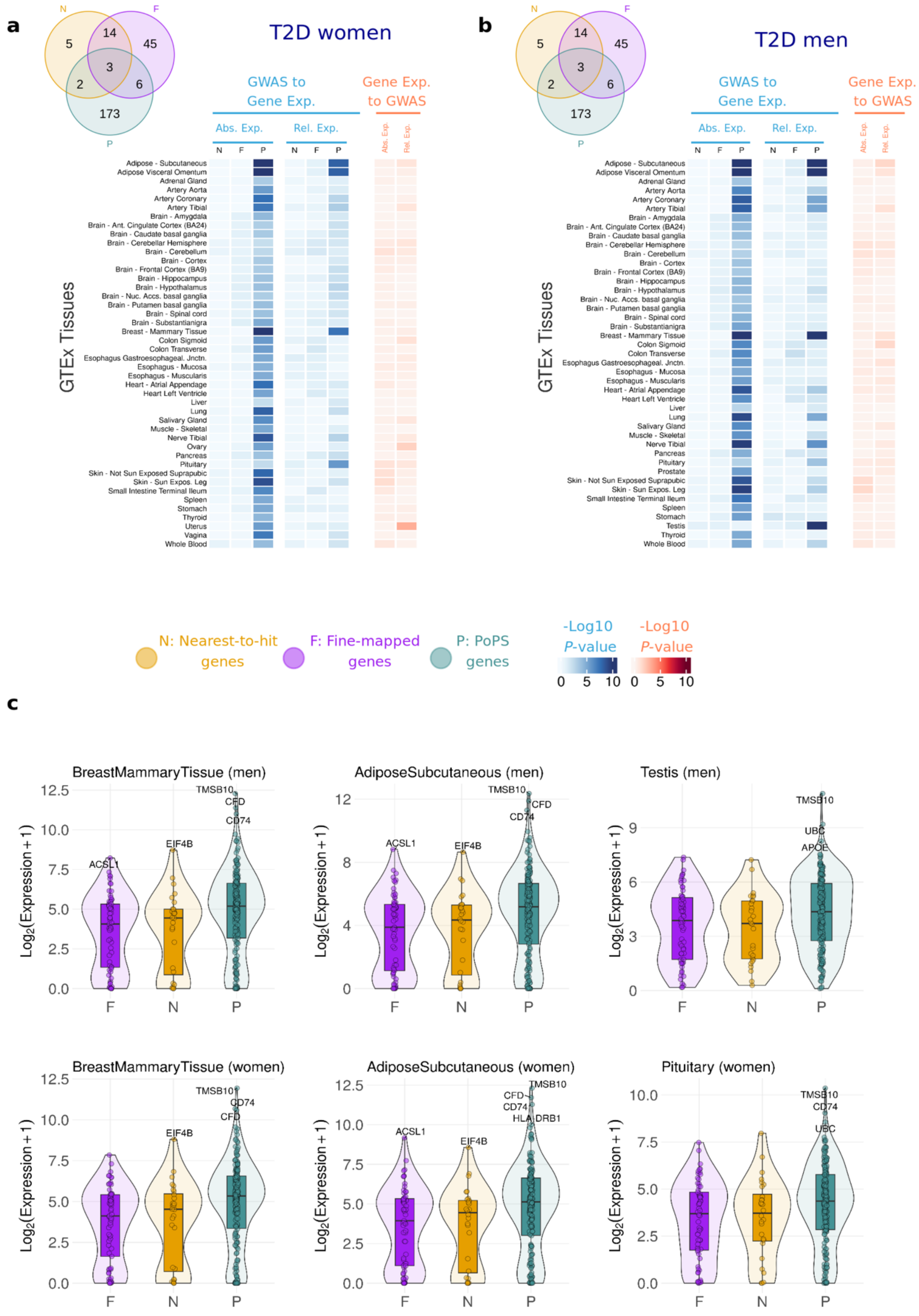

Supplemental Figure 13: Sex-stratified analyses for T2D.

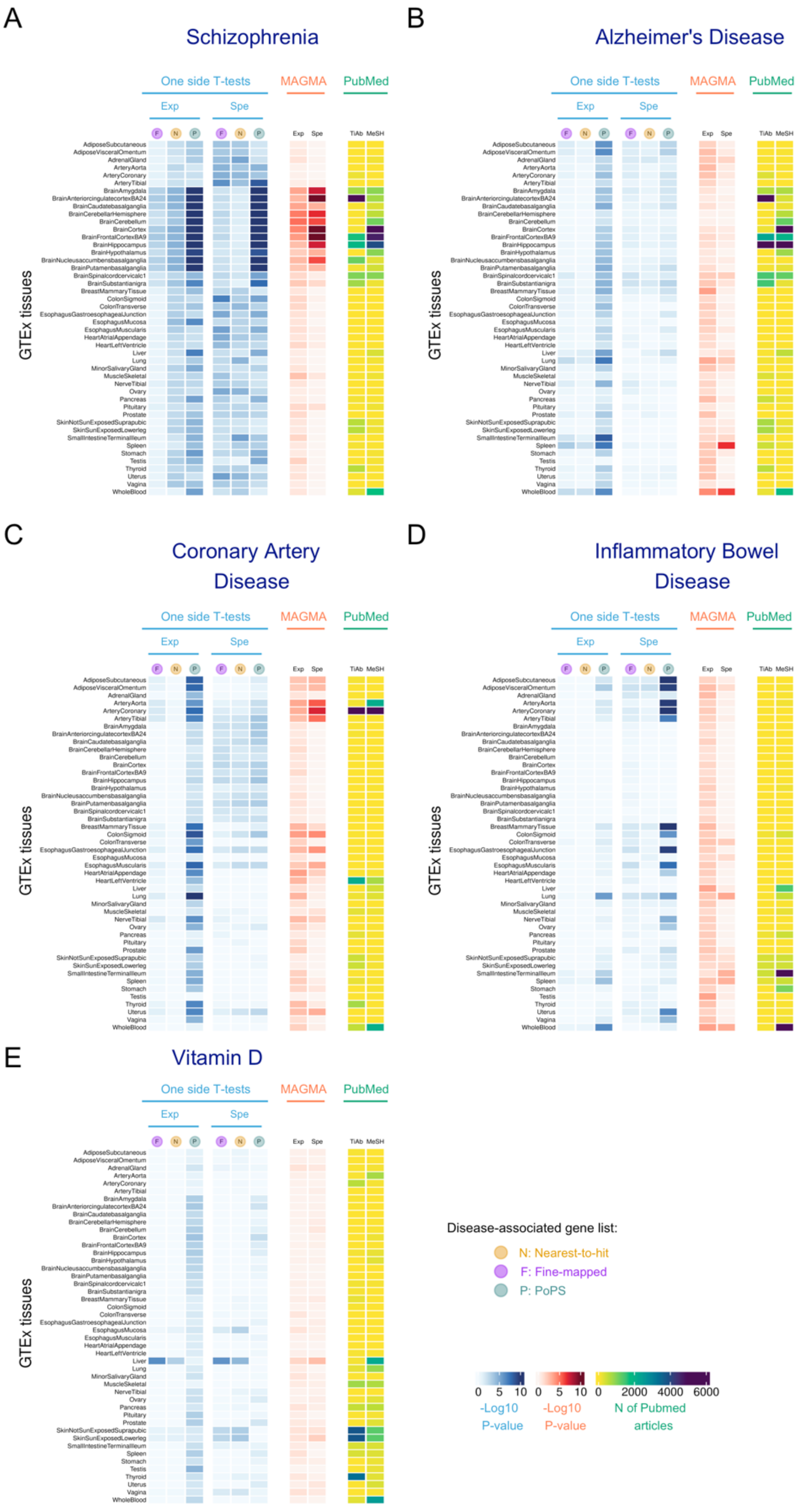

Supplemental Figure 14. Heatmap showing results of the sensitivity analysis where association between gene expression in each GTEx tissue was performed after removal of the top 10% genes most expressed or most specifically expressed in relevant tissues.

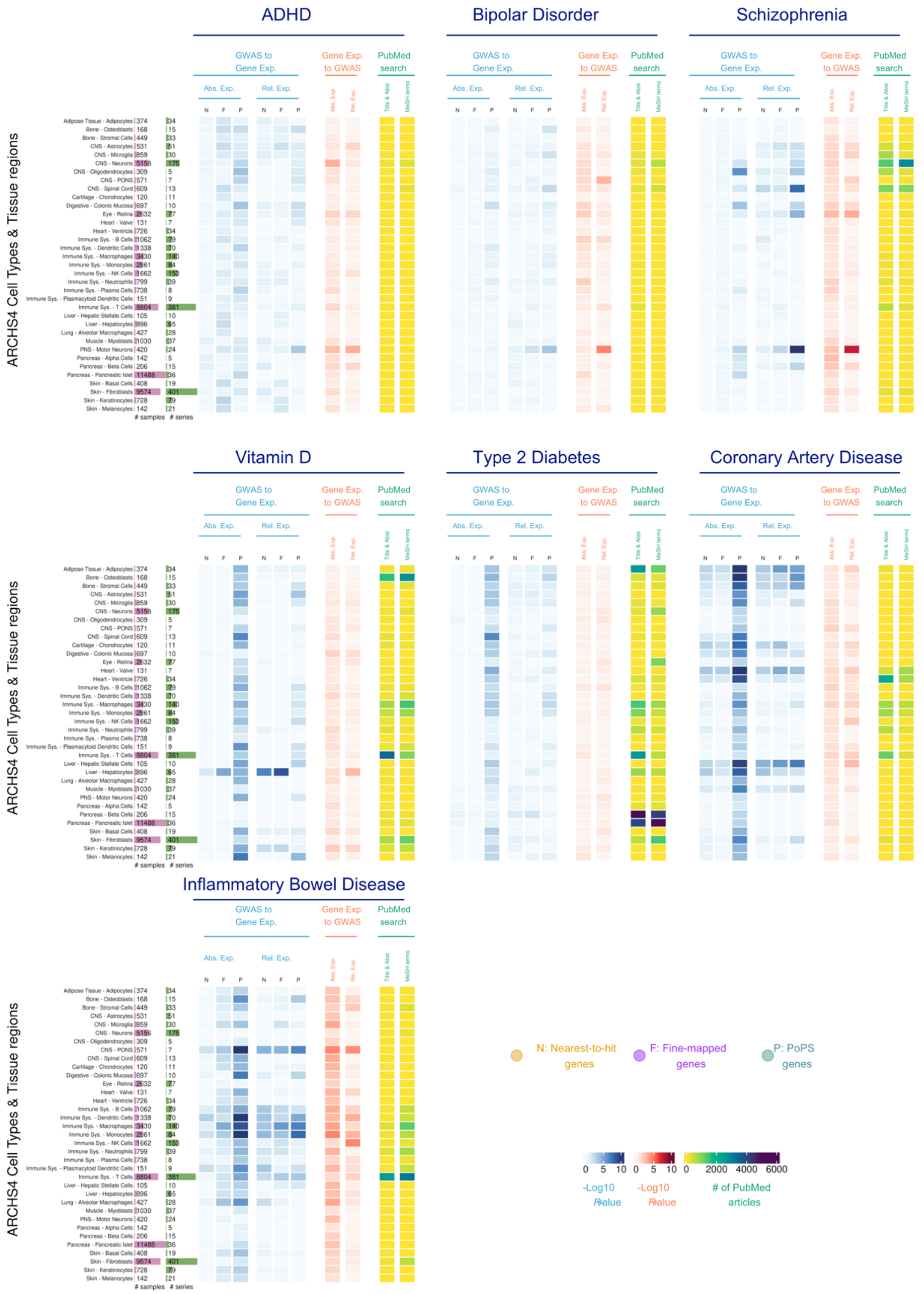

Supplemental Figure 15: Heatmap showing results of the association between gene expression in each cell-type or tissue region and ADHD, Bipolar Disorder, Schizophrenia, Vitamin D, and Type 2 Diabetes **using the ARCHS4 dataset**. In blue, results showing the Log_{10} *P*-value for a one-side T-tests. In red, results showing the Log_{10} *P*-value for enrichment of GWAS signal across the set of genes with highest absolute and relative expression for each tissue. In yellow, results for the Literature Search using PubMed. Abs. Exp, Absolute expression; Spec. Exp, specific expression; F, Fine-mapped genes; N, Nearest-to-hit genes, P, Polygenic Priority Scores genes; Title & Abst, Title and Abstract are used in the PubMed Search; MeSH, MeSH terms are used in the PubMed Search.

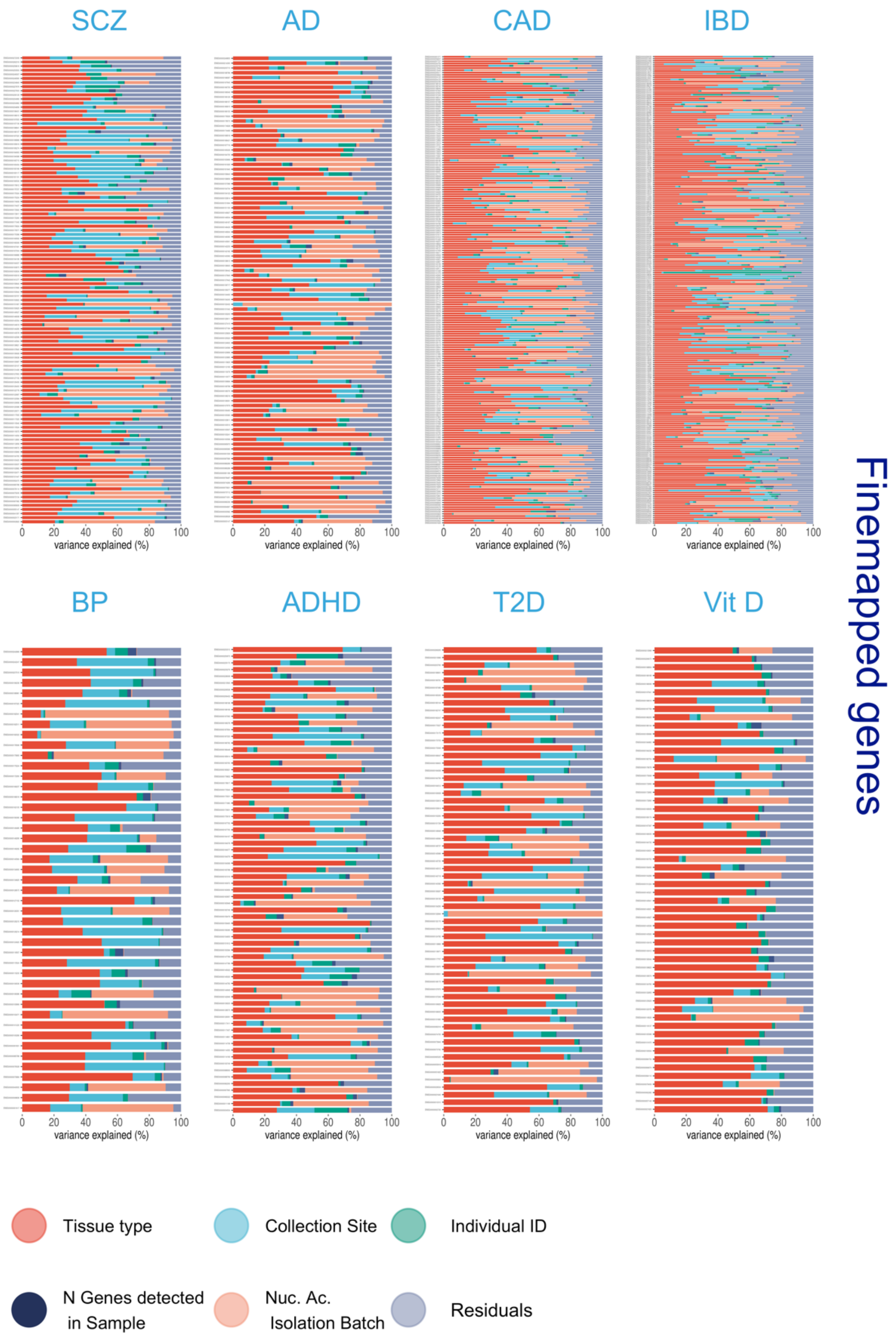

Supplemental Figure 16. Factors explaining gene expression variance in disease-associated genes prioritized via fine-mapping of GWAS hits. Each percentage bar (each row) corresponds to an individual gene. Panels are split by prioritization method and trait investigated.

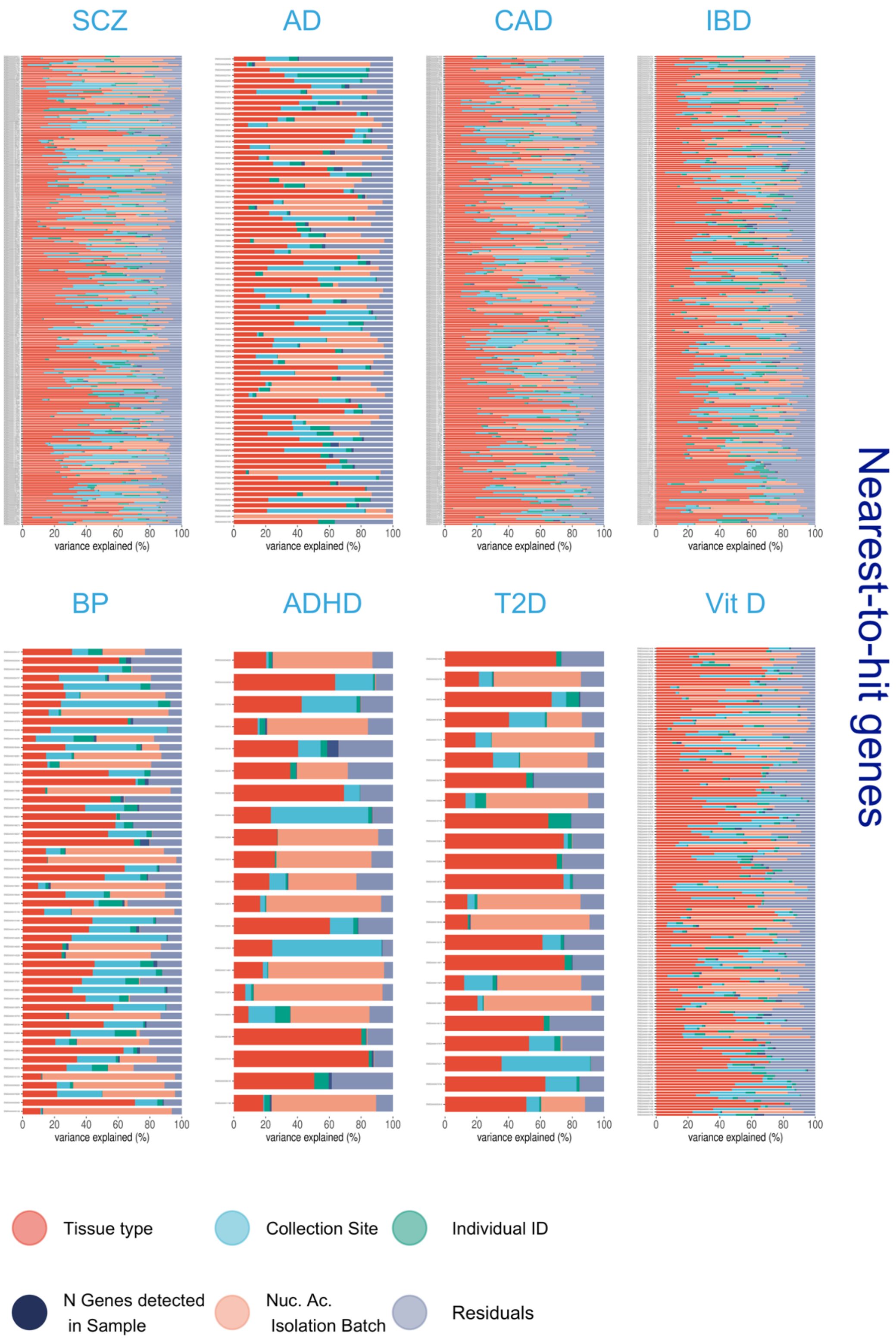

Supplemental Figure 17. Factors explaining gene expression variance in disease-associated genes prioritized using the nearest to GWAS-hit approach. Each percentage bar (each row) corresponds to an individual gene. Panels are split by prioritization method and trait investigated.

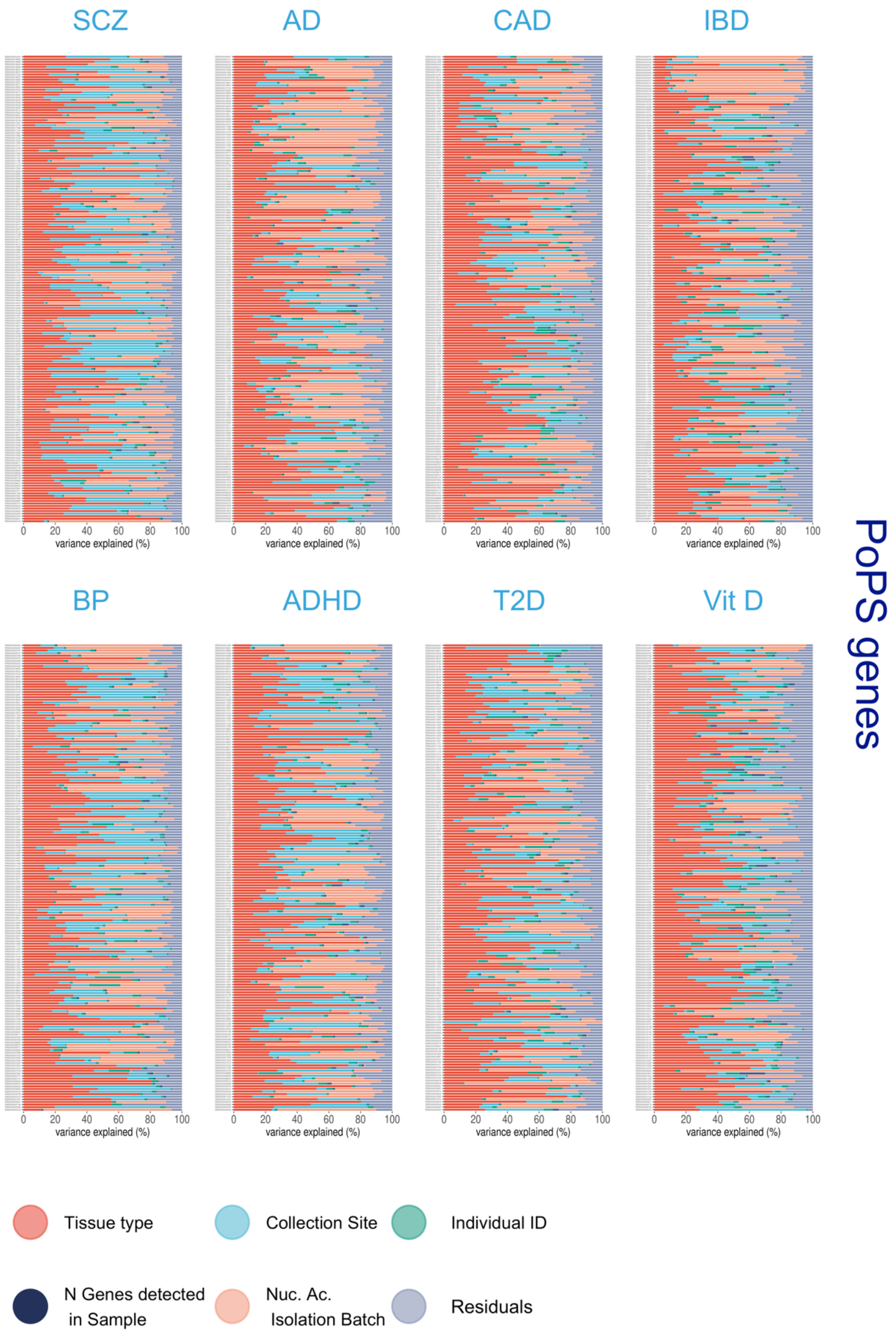

Supplemental Figure 18. Factors explaining gene expression variance in disease-associated genes using the PoPS method. Each percentage bar (each row) corresponds to an individual gene. Panels are split by prioritization method and trait investigated.

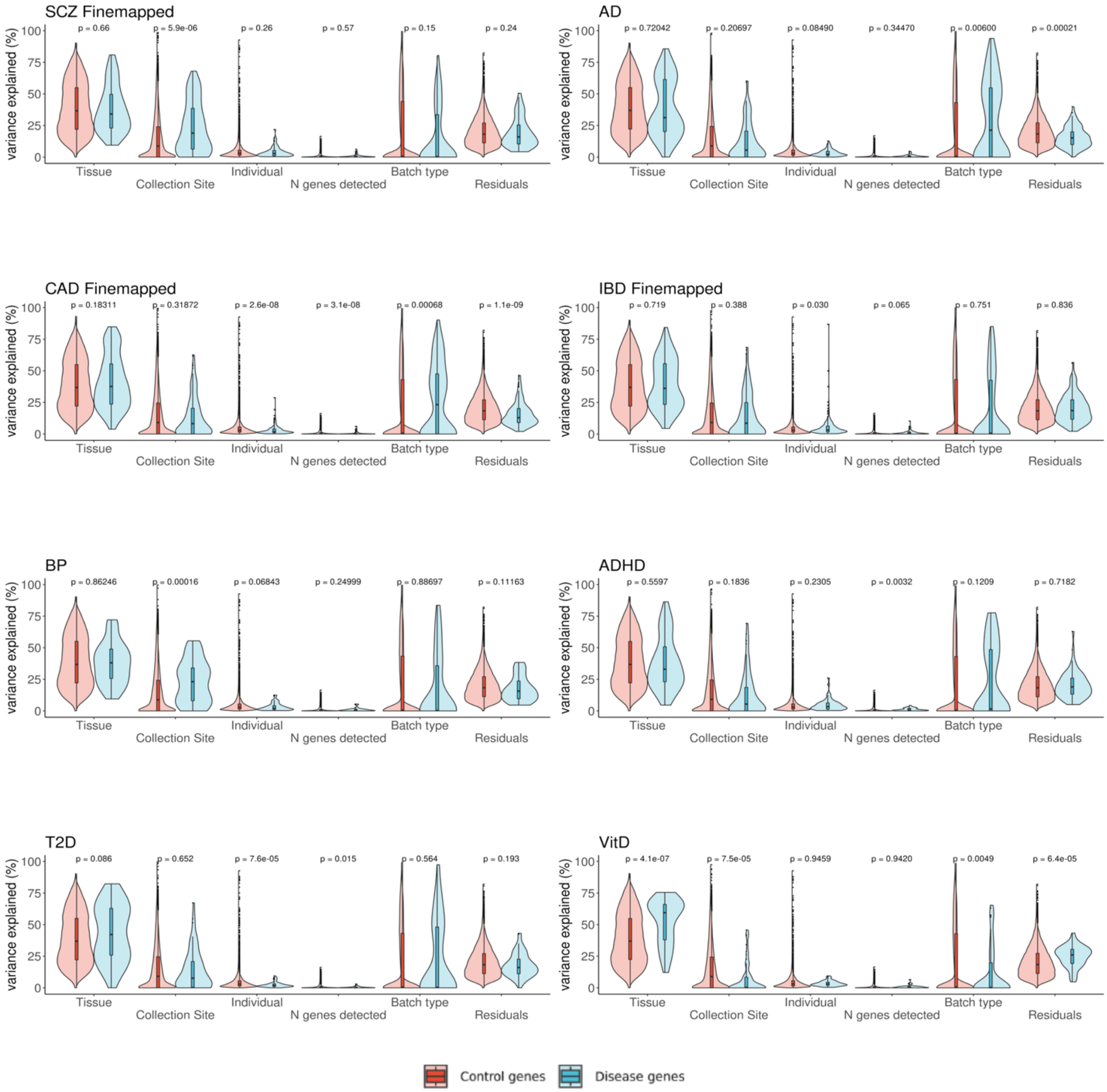

Supplemental Figure 19. Factors explaining gene expression in disease-associated genes via *Finemapping* vs other protein-coding genes. Every data point composing the violin plots represents the proportion of variance explained by a predictor (Tissue, Collection Site, Individual N genes detected, Batch Type) on the gene expression of an individual gene. This is calculated by fitting a linear mixed model for each gene. The results are color-coded to compare the variance explained by each factor in fine-mapped genes vs control genes.

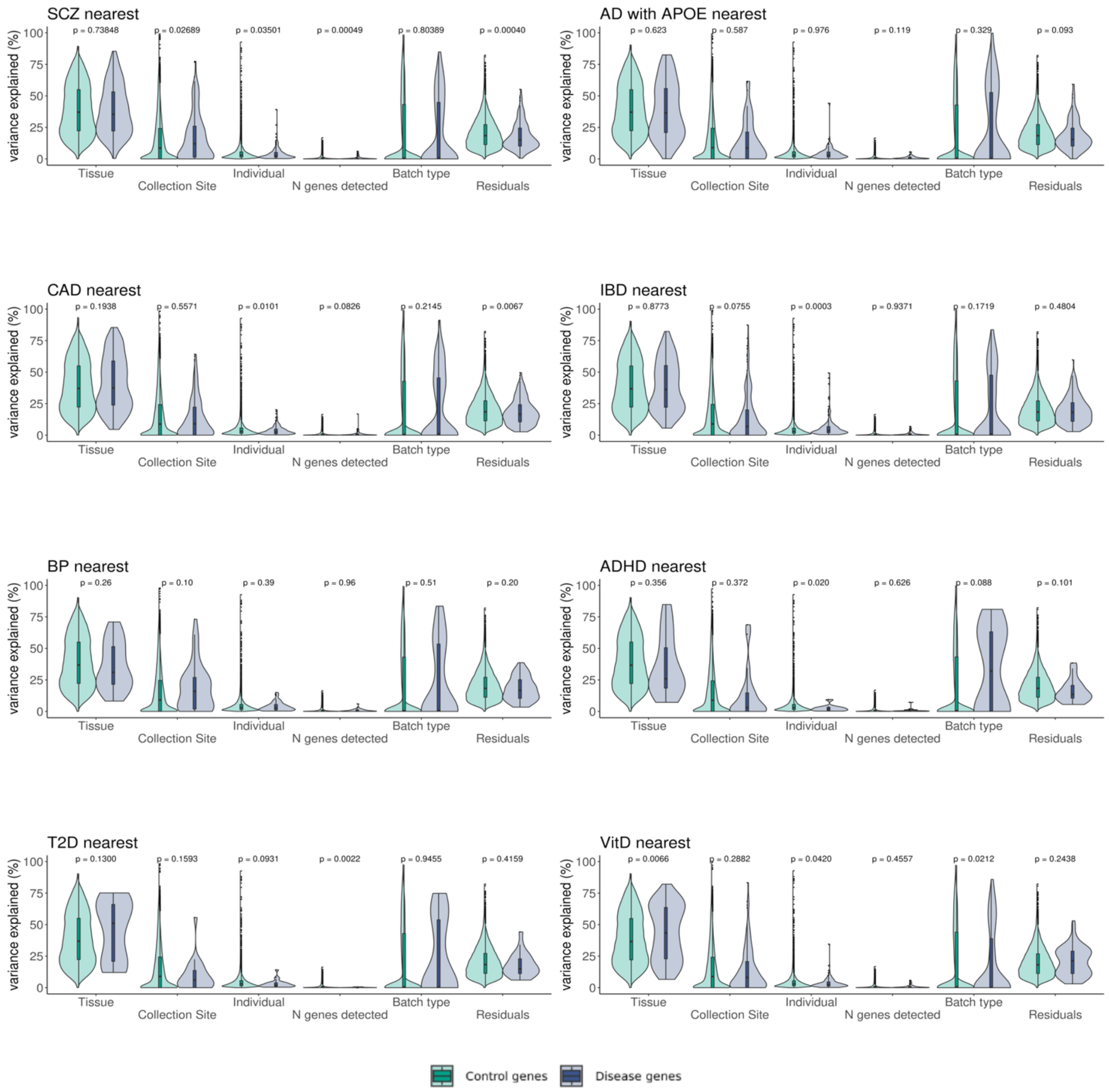

Supplemental Figure 20. Factors explaining gene expression in disease-associated genes via *nearest genomic location* vs other protein-coding genes. Every data point composing the violin plots represents the proportion of variance explained by a predictor (Tissue, Collection Site, Individual N genes detected, Batch Type) on the gene expression of an individual gene. This is calculated by fitting a linear mixed model for each gene. The results are color-coded to compare the variance explained by each factor in *nearest-to-GWAS-hit* genes vs control genes.

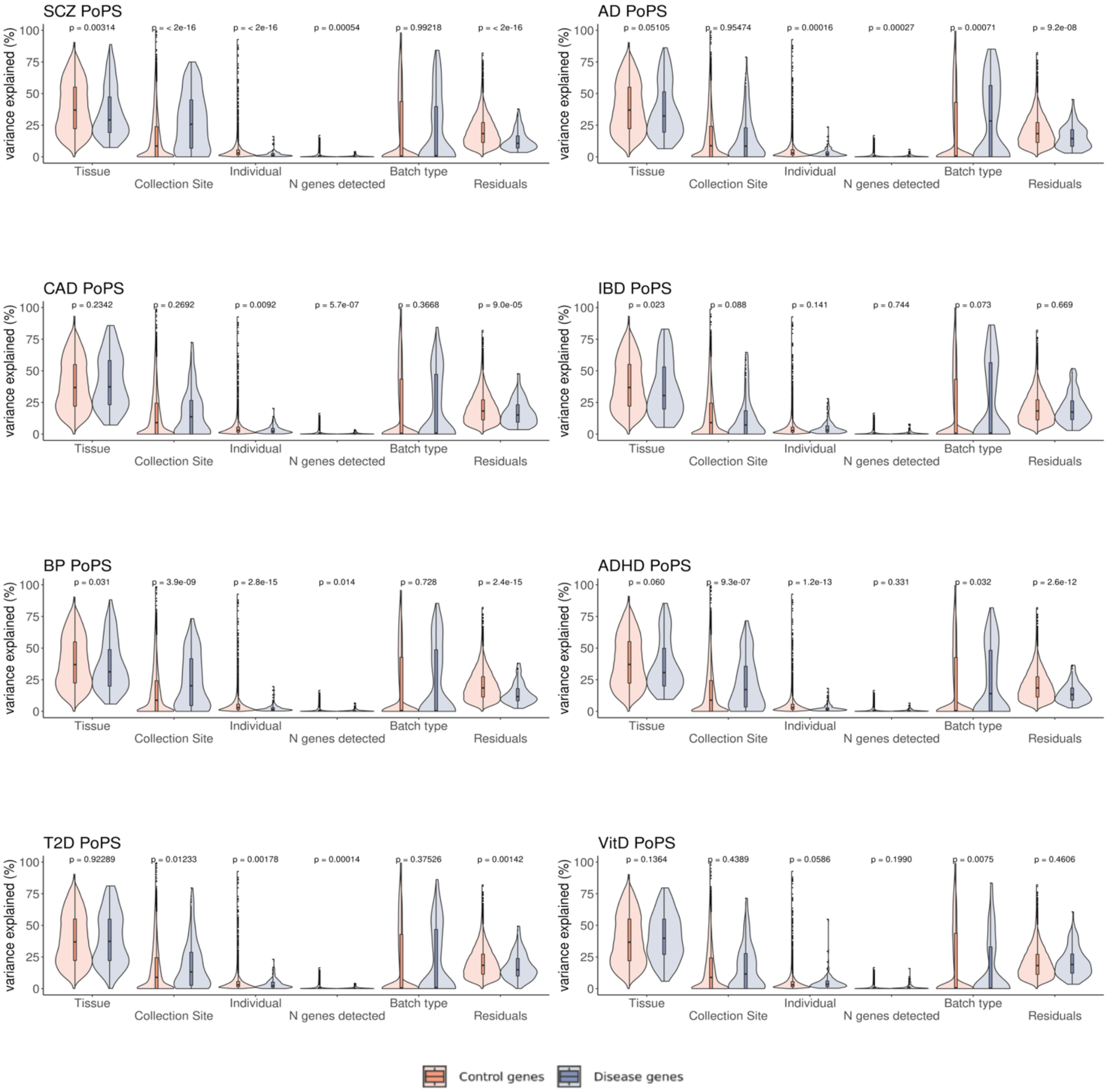

Supplemental Figure 21. Factors explaining gene expression in disease-associated genes via the PoPS prioritization method vs other protein-coding genes. Every data point composing the violin plots represents the proportion of variance explained by a predictor (Tissue, Collection Site, Individual N genes detected, Batch Type) on the gene expression of an individual gene. This is calculated by fitting a linear mixed model for each gene. The results are color-coded to compare the variance explained by each factor in *PoPS* genes vs control genes.

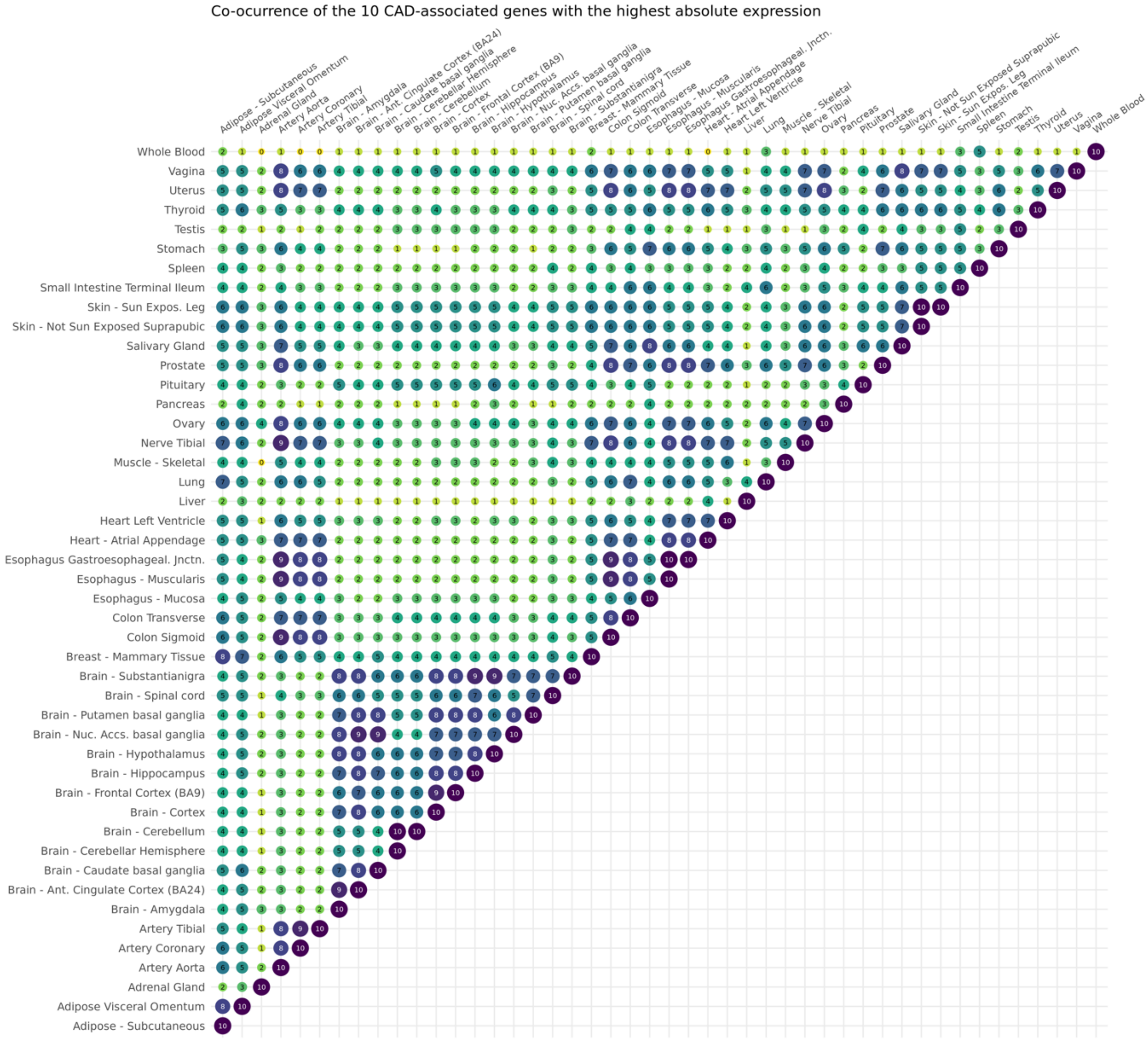

Supplemental Figure 22. Co-occurrence of the 10 CAD most expressed, CAD associated genes across GTEx tissues.

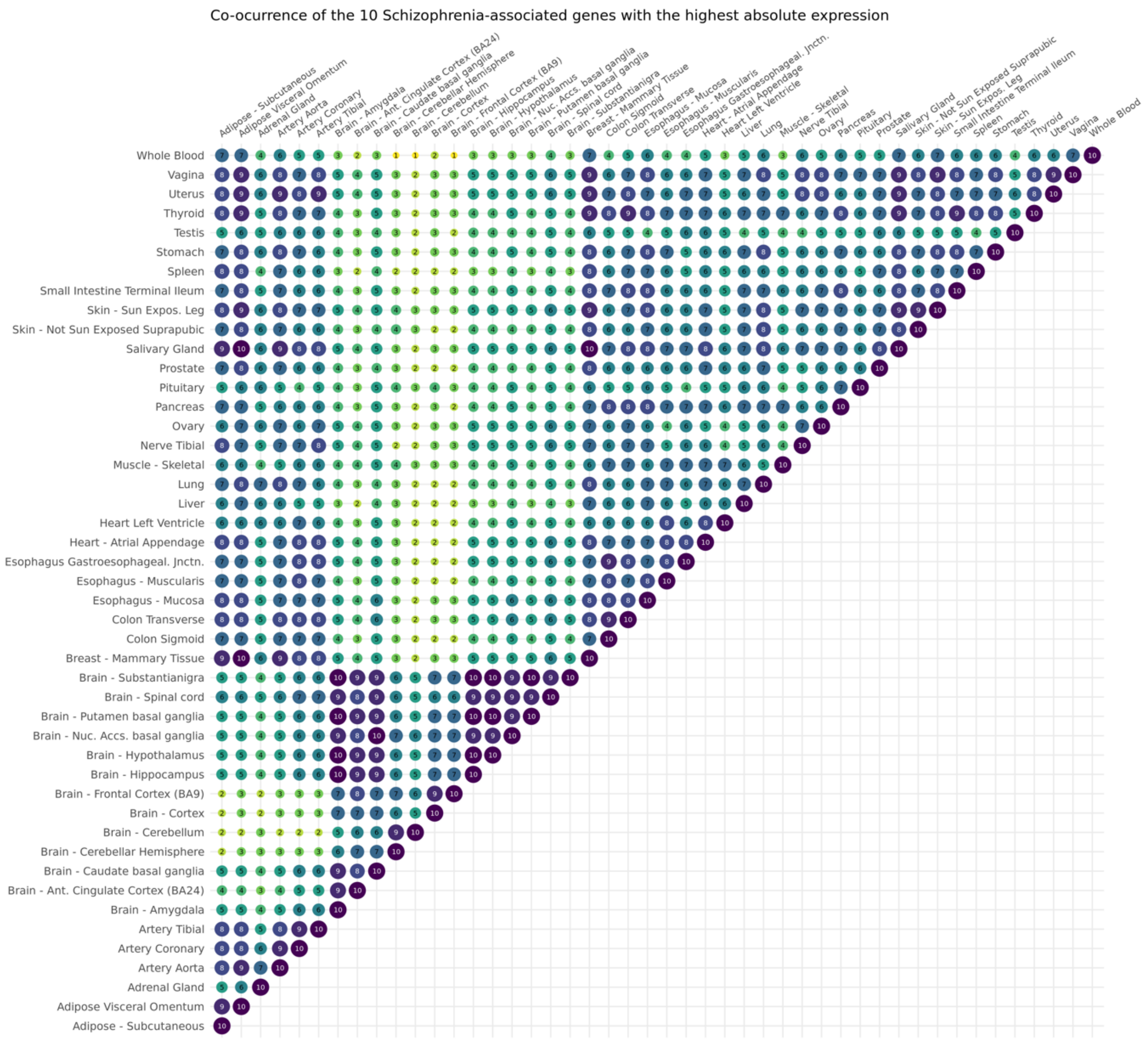

Supplemental Figure 23. Co-occurrence of the 10 most expressed, schizophrenia associated genes across GTEx tissues.

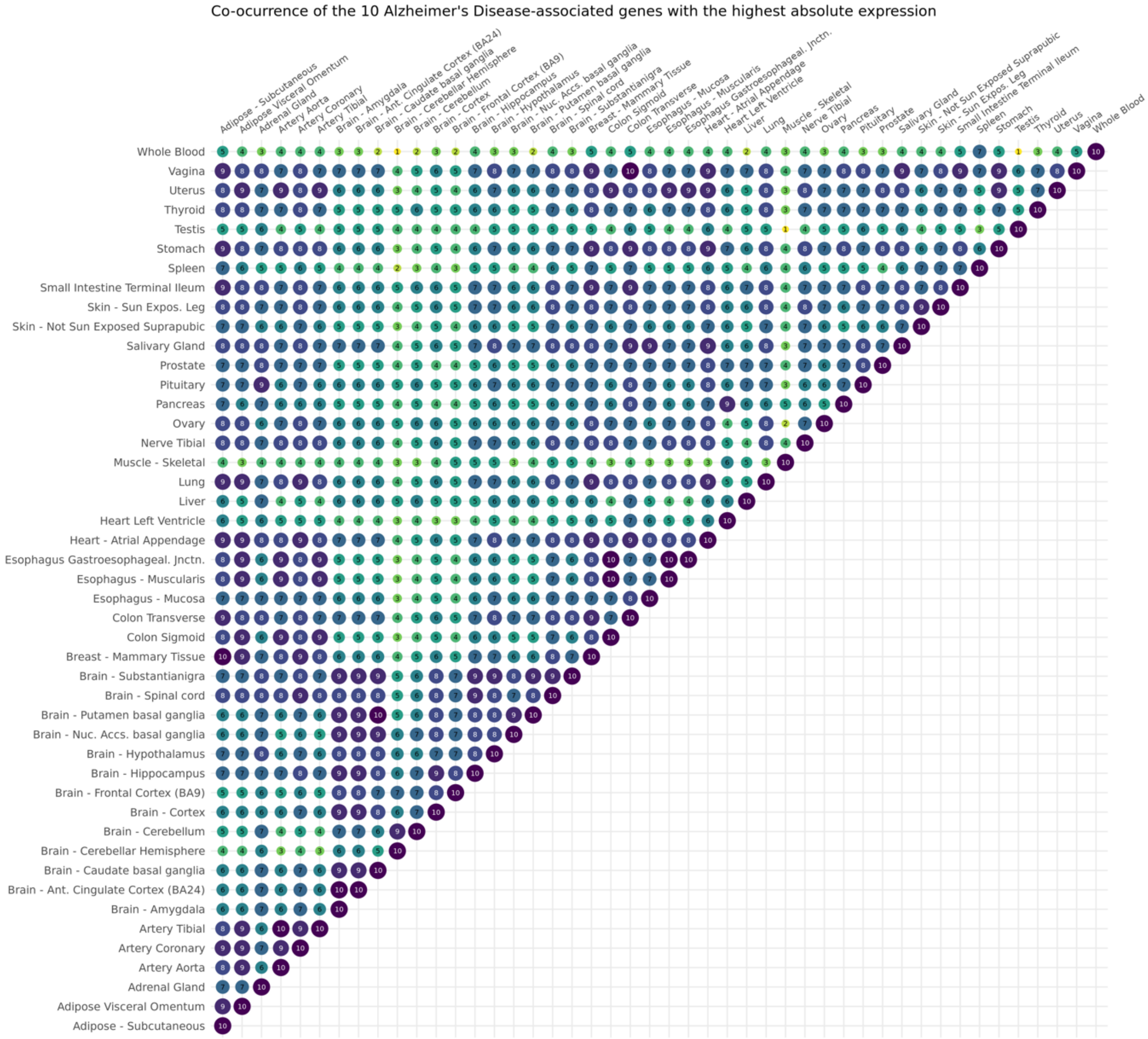

Supplemental Figure 24. Co-occurrence of the 10 most expressed, Alzheimer's disease associated genes across GTEx tissues.

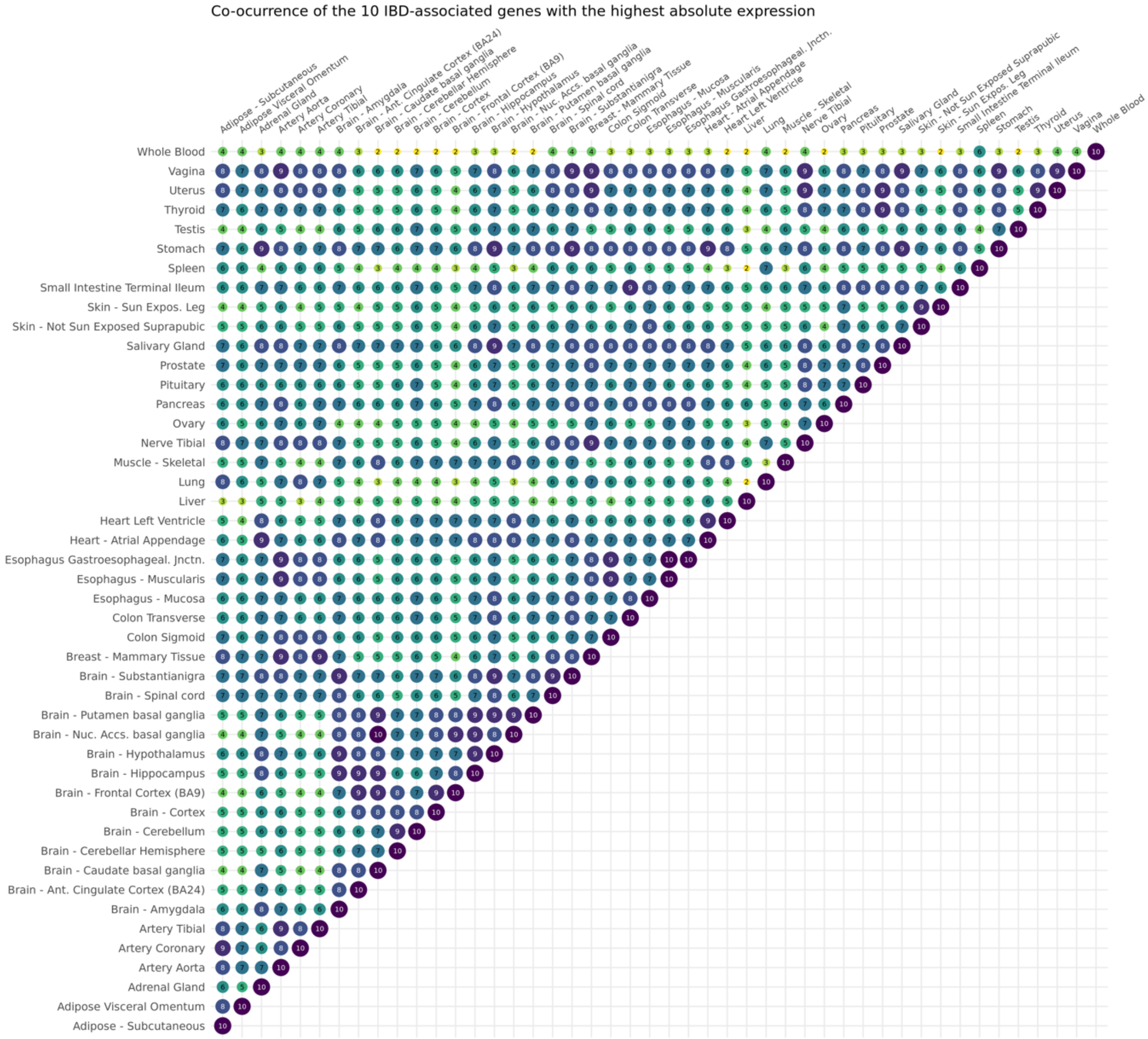

Supplemental Figure 25. Co-occurrence of the 10 most expressed, Inflammatory Bowel Disease associated genes across GTEx tissues.

Co-ocurrence of the 10 Bipolar Disorder-associated genes with the highest absolute expression

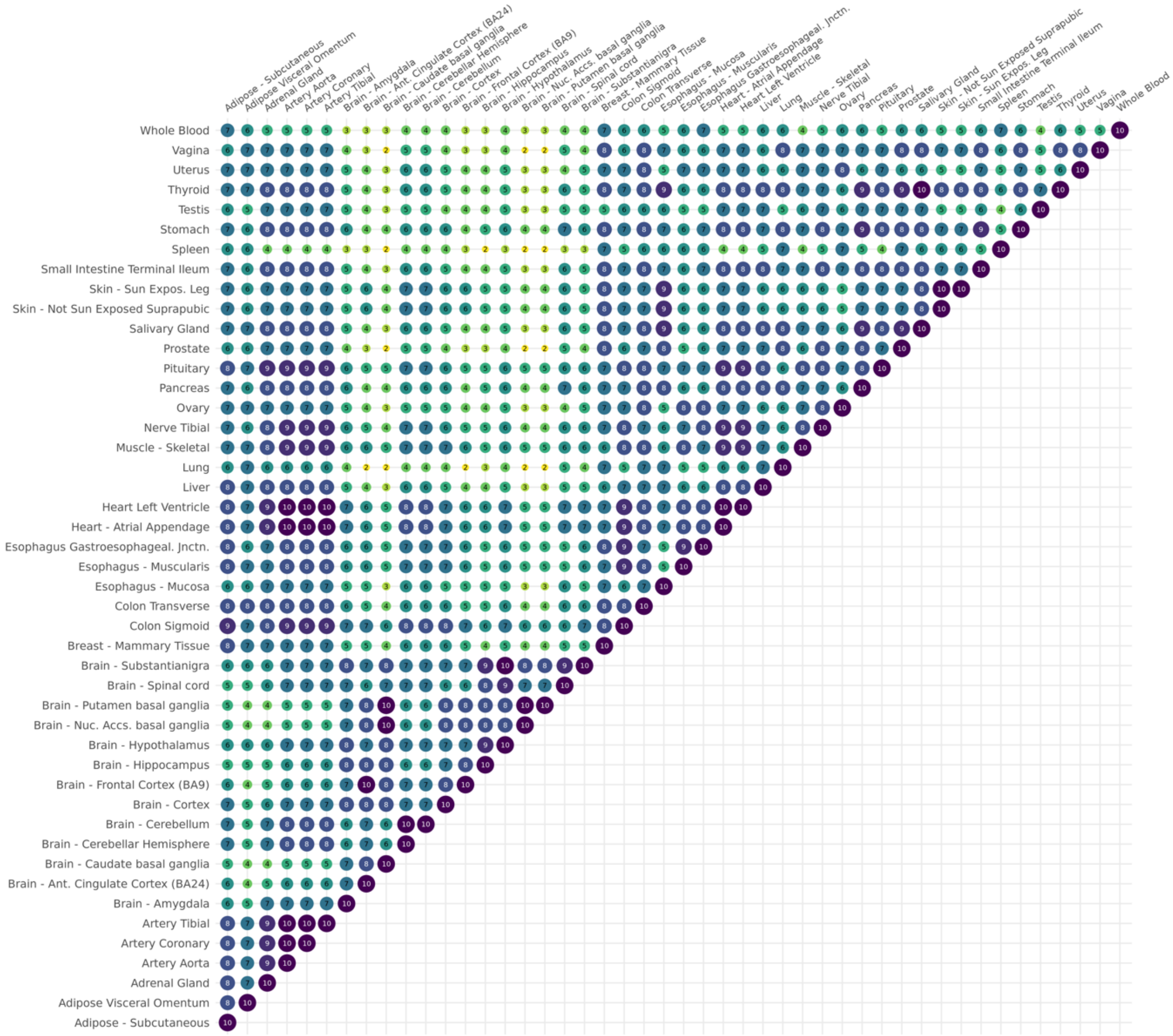

Supplemental Figure 26. Co-occurrence of the 10 most expressed, Bipolar Disorder associated genes across GTEx tissues

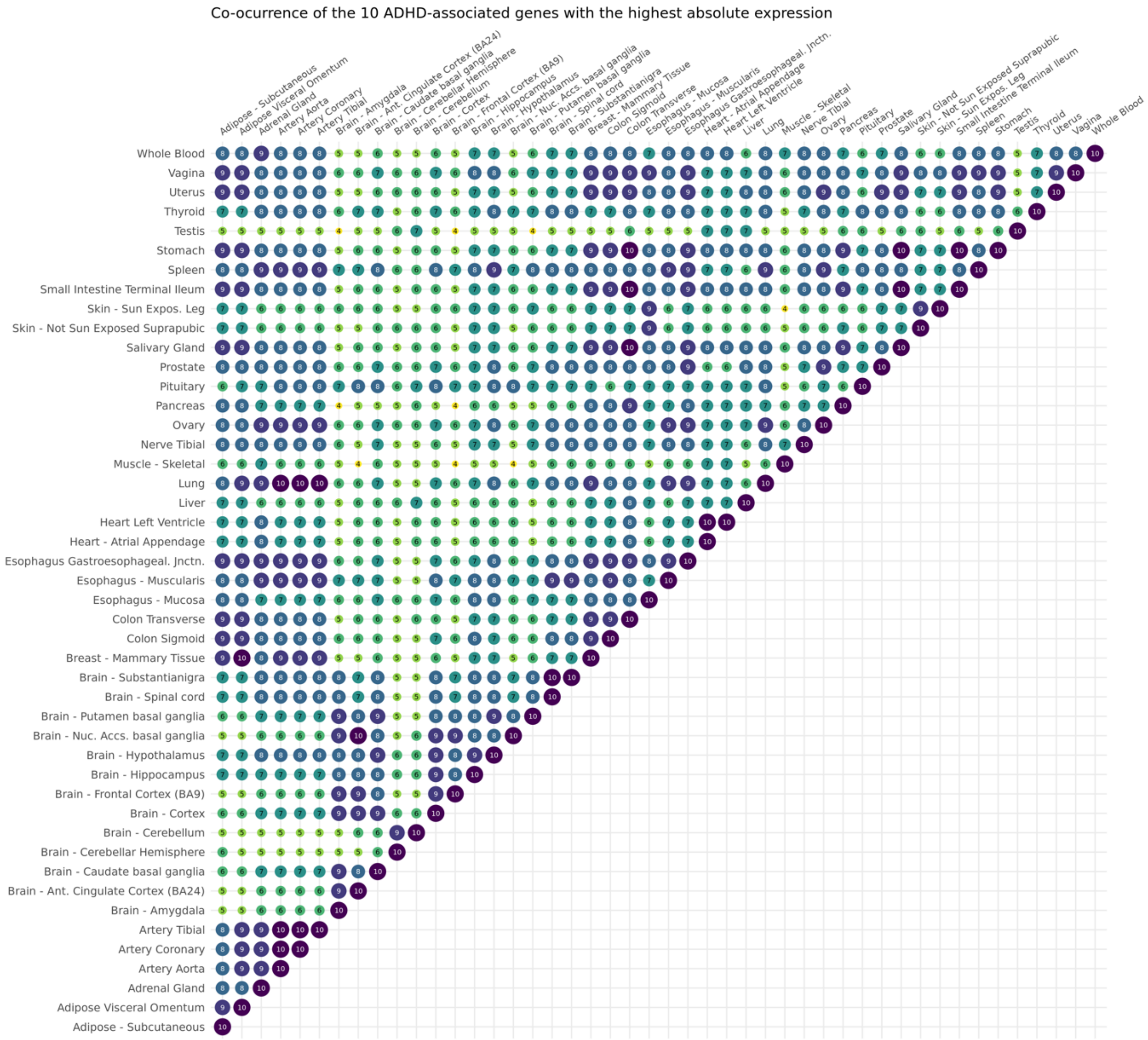

Supplemental Figure 27. Co-occurrence of the 10 most expressed, ADHD associated genes across GTEx tissues

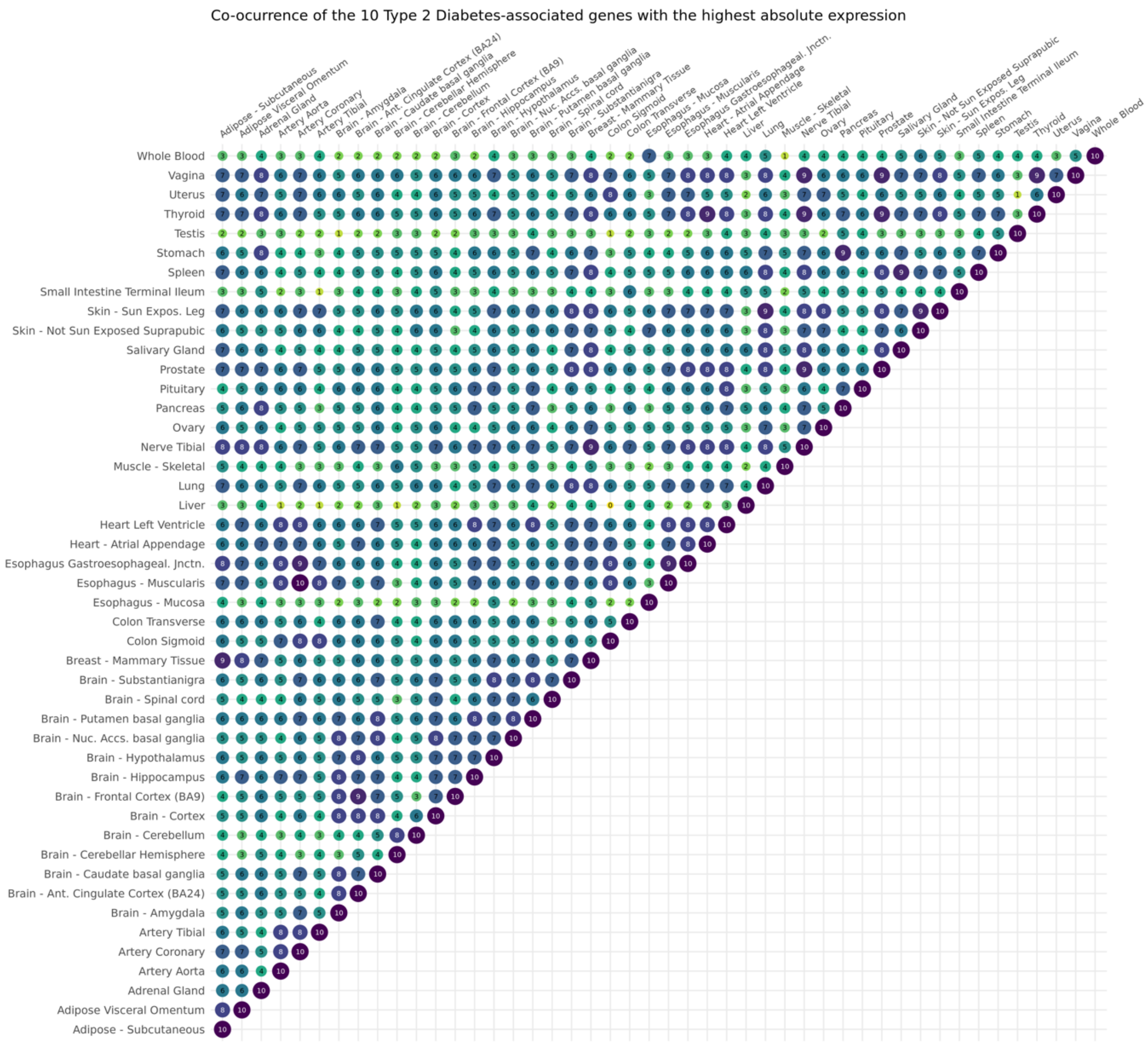

Supplemental Figure 28. Co-occurrence of the 10 most expressed, Type 2 Diabetes associated genes across GTEx tissues

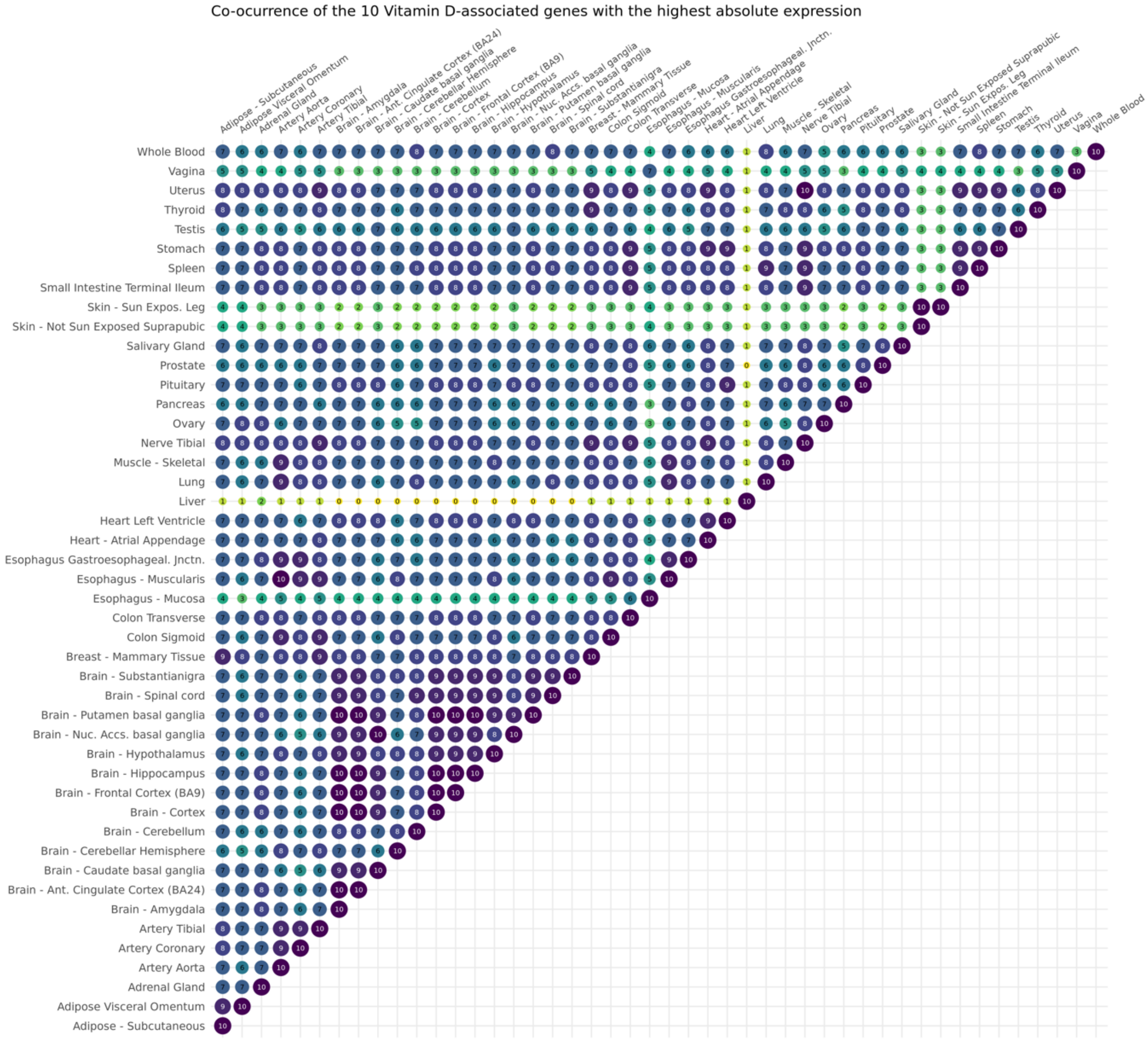

Supplemental Figure 29. Co-occurrence of the 10 most expressed, Vitamin D associated genes across GTEx tissues
